# Social, environmental, and developmental factors affect the microbiota of barn owls (*Tyto alba*) in a cross-fostering experiment

**DOI:** 10.1186/s42523-024-00365-w

**Published:** 2024-12-24

**Authors:** Ammon Corl, Motti Charter, Gabe Rozman, Sondra Turjeman, Sivan Toledo, Pauline L. Kamath, Wayne M. Getz, Ran Nathan, Rauri C. K. Bowie

**Affiliations:** 1 Museum of Vertebrate Zoology, University of California, Berkeley, 3101 Valley Life Sciences Building, Berkeley, CA 94720-3160 USA; 2 Department of Integrative Biology, University of California, Berkeley, 3060 Valley Life Sciences Building, Berkeley, CA 94720 USA; 3 The Shamir Research Institute, Department of Geography and Environmental Studies, University of Haifa, 199 Aba Hushi Boulevard, Mount Carmel, Haifa, 3498838 Israel; 4 Movement Ecology Lab, Department of Ecology, Evolution, and Behavior, Alexander Silberman Institute of Life Sciences, The Hebrew University of Jerusalem, Edmond J. Safra Campus at Givat Ram, Jerusalem, 91904 Israel; 5 Azrieli Faculty of Medicine, Bar-Ilan University, Safed, 1311502 Israel; 6 Blavatnik School of Computer Science, Tel Aviv University, Tel Aviv, Israel; 7 School of Food and Agriculture, University of Maine, 5735 Hitchner Hall, Orono, ME 04469 USA; 8 Maine Center for Genetics in the Environment, University of Maine, 5703 Alumni Hall, Orono, ME 04469 USA; 9 Environmental Science, Policy, and Management, University of California, Berkeley, VLSB 5048-B, Berkeley, CA 94720 USA; 10 School of Mathematical Sciences, University of KwaZulu, Natal, South Africa

**Keywords:** Microbiota, Cross-fostering, Development, Movement ecology, Social transmission, *Tyto alba*

## Abstract

**Background:**

Species host diverse microbial communities that can impact their digestion and health, which has led to much interest in understanding the factors that influence their microbiota. We studied the developmental, environmental, and social factors that influence the microbiota of nestling barn owls (*Tyto alba*) through a partial cross-fostering experiment that manipulated the social and nest environment of the nestlings. We then examined the nestling microbiota before and three weeks after the exchange of nestlings between nests, along with the microbiota of the adults at the nest and nestlings in unmanipulated nests.

**Results:**

We found that nestlings had higher bacterial diversity and different bacterial communities than adults. The microbiota of nestlings was more like that of their mothers than their fathers, but the similarity to the father tended to increase with the amount of time the father was in close proximity to the nest, as measured from movement data. Cross-fostered offspring had higher bacterial diversity and greater changes in bacterial community composition over time than control offspring. Cross-fostering led the microbiota of the nestlings in the experiment to converge on similar bacterial communities. The microbiota of nestling owls therefore rapidly changed along with alterations to their social and nest environments.

**Conclusions:**

These results highlight the dynamic nature of the microbiota during early development and that social interactions can shape microbial communities.

**Supplementary Information:**

The online version contains supplementary material available at 10.1186/s42523-024-00365-w.

## Background

Animals are home to diverse microbial communities that can interact with the host species in many important ways [1–4]. Some bacteria aid in digestion or detoxify compounds in the food [5–7]. The microbiota of hosts can harbor pathogens [8, 9], but can also help prevent pathogens from colonizing the host [10–12], both of which impact host health. Some bacteria form tight symbioses with host species [13] and can affect host development, behavior, and survival [4, 14]. These ecologically important interactions between hosts and their microbiota have generated considerable interest in exploring the factors that shape the microbial communities of hosts [15–17].

The microbiota associated with hosts are influenced by many factors including the hosts’ physical environment [18], diet [19–21], behavior [22–24], physiology [25], and genetics [26]. One way to explore the influence of these factors is to study the early development of the microbiota. Large changes in the microbiota have been observed in early development as animals age [27–31]. Changes in the microbiota could be driven by changes in food sources as animals mature, the development of the host immune system, the selection over time of microbes that thrive in the host from the larger microbial community, or competitive interactions among members of the microbiota [4, 32, 33]. This process of establishing a microbiota can impact the health, growth, and fitness of the host [4, 10, 12].

Social transmission of bacteria among hosts can shape the composition and diversity of the microbiota found within hosts. Microbes can disperse among closely interacting individuals (e.g. mates, parents and offspring, predators and prey, grooming partners) [18, 34–37], which can lead to the homogenization of microbiota among interacting hosts [17, 28, 38–40]. In some cases, direct contact between hosts is not even necessary for bacteria to spread between hosts inhabiting the same environment [41, 42]. Bacterial taxa can differ in their modes of social transmission, because in mice obligate aerobes tend to be transmitted horizontally and obligate anaerobes tend to be transmitted vertically [41] and in baboons socially structured bacteria tend to be anaerobic and non-spore forming [37]. Social transmission of bacteria among hosts sometimes has stronger effects on the microbiota than genetic differences [38] or species differences in microbial communities [28, 43], although these effects can be transitory [28]. Thus, social effects on the microbiota are likely to be important, but need to be studied through detailed sampling or experimental manipulations that separate out social factors from dietary, genetic, and environmental factors shared among socially interacting individuals [16].

A powerful way to study the establishment of a host’s microbiota is to cross-foster offspring between families, which experimentally manipulates the early environment of the offspring. Cross-fostering can help separate out the influence of genetic, social, and environmental factors in the development of organismal traits [44, 45]. Cross-fostering offers a rare opportunity to not only observe the microbiota of non-model species, but to experimentally manipulate factors that could affect their microbiota [28, 39, 43, 46, 47]. Cross-fostering experiments have revealed that the microbiota of cross-fostered offspring can quickly change to resemble their foster siblings in both intraspecific [34, 39] and interspecific [43] exchanges of offspring. However, other cross-fostering experiments suggest that the original environment can have lasting effects on the microbiota of cross-fostered offspring [47] and that host species identity can be important for structuring the microbiota [28, 46]. Therefore, additional studies are needed to help reveal the factors that dictate whether a host’s microbiota is maintained or changed in new environmental conditions.

We conducted a partial cross-fostering experiment with nestling barn owls (*Tyto alba*) to investigate factors that affect their microbiota. Our study also includes microbiota data from the nestlings’ parents, many of which were studied previously [48], which provides a foundation of knowledge about the microbiota of these owls. For example, we previously found that adult female owls had greater bacterial diversity and different bacterial communities than males. For both sexes, owls that traveled greater distances away from their nests had higher bacterial diversity. The adult owls’ bacterial diversity was also correlated with the age of the oldest nestling, clutch size, and fledgling success.

Our prior work on adult barn owls inspired us to study whether the microbiota of barn owl nestlings is affected by age, sex differences, and the movement behavior of their parents. Many avian studies have observed differences in the microbiota of adults and their offspring [27–30], which suggested that it could be an important factor to examine in our barn owl system. Males and females could differ in their microbiota because of sexual differences in physiology, behavior or diet. For example, male and female mice have different microbiota because of differences in sex hormones and immunological responses [49–51]. Although sex differences in the microbiota have been observed in some studies of birds [23, 25, 52], other avian studies have not found such a pattern [15, 22, 53, 54]. These studies were typically done on adult birds, with the one exception demonstrating that the microbiota of nestlings may not exhibit the sex differences observed in adults [52], which suggests that further studies are warranted. Similarly, there have been few studies that have examined host microbiota in relation to their movement behavior [22–24, 35]. However, these studies have demonstrated that movement behavior can be important, because differences in the microbiota have been associated with migratory behavior [22–24] and social interactions that result in bodily contact [35].

Our goal was to investigate the developmental, environmental, and social factors that may affect the host-associated microbiota by studying barn owl nestlings and their parents. This was accomplished through a partial cross-fostering experiment in which approximately equal numbers of offspring of the same age were either swapped between nests (cross-fostered) or kept at their natal nest (controls). We collected data on the microbiota of the control and cross-fostered nestlings, unmanipulated nestlings not involved in the cross-fostering experiment, and the adult owls at the experimental and unmanipulated nests. We had three major lines of inquiry: (1) comparisons across different age classes and between the sexes, (2) comparisons among nestlings in the experimental groups, and (3) comparisons of nestlings to their parents. We considered not only the effects of the parents and natal environment as is done in a typical cross-fostering experiment, but also the potential for horizontal transmission of microbes among nestlings that are cross-fostered together. We tested the following eight hypotheses regarding factors that could influence the nestlings’ microbiota.

### Comparisons between the sexes and across different age classes

*Developmental Changes Hypothesis.* The microbiota changes over developmental stages, because of changes in the immune system, exposure to new bacteria, or other factors. Therefore, we hypothesize that young nestlings, old nestlings, and adults could exhibit differences in their microbiota in terms of bacterial species richness (i.e. alpha diversity), bacterial community compositions (i.e. beta diversity), or both alpha and beta diversity. Studies of other birds suggest that adults and offspring often have differences in both alpha and beta diversity, but offspring may have either higher or lower bacterial species richness than their parents [27–30].

*Sexual Differentiation Hypothesis.* Male and female nestlings will have different microbiota, given the sex differences observed in adult owls [48]. Alternatively, sex differences in the microbiota may develop later in life and only be observed in adult owls.

### Comparisons among nestlings

*Increased Alpha Diversity Hypothesis.* Cross-fostered nestlings, unlike control nestlings, are exposed to two different nest environments and pairs of adults, so will have higher bacterial species richness (i.e. alpha diversity).

*Increased Microbiota Change Hypothesis.* Cross-fostered offspring will show greater changes in their microbiota over time than control offspring because of exposure to two social and nest environments rather than just one.

*Convergent Bacterial Community Compositions (i.e. Beta Diversity) Hypothesis.* The bacterial communities of cross-fostered offspring will come to resemble their adopted siblings due to sharing a common nest environment and from bacterial transfer from their adopted family. Cross-fostered offspring may still share bacteria with their biological siblings but are expected to gain new bacteria that increase their similarity to their adopted siblings. We would reject this hypothesis if cross-fostered nestlings do not match their adopted siblings, which could occur if their microbiota is slow to change, not affected by their environment, or dictated by genetic, developmental, or random factors.

### Comparisons of nestlings to parents

*Higher Similarity to Mothers than Fathers Hypothesis.* When nestlings are young, barn owl mothers remain at the nest, while fathers hunt and bring prey to the mother who feeds the nestlings [55]. When nestlings are ~ 24 days old, the mother resumes hunting and both parents may feed the nestlings. We hypothesized that nestlings would show greater similarity to their mothers than to their fathers in alpha or beta diversity (or both) because of their shared nest environment and the greater potential for social transmission of microbes between mothers and offspring during their early development.

*Reduced Similarity to Parents with Cross-fostering Hypothesis.* Compared to control offspring, cross-fostered offspring are predicted to have decreased similarity to their biological parents because they were moved to a different nest environment with new siblings and adults.

*Parental Movement Ecology affects Microbial Similarity to Offspring Hypothesis.* Parents that spend more time at their nests will have greater similarity to their offspring’s microbiota because there is more opportunity for social transmission of microbes.

## Methods

### Owl cross-fostering and data collection

**Fig. 1:**
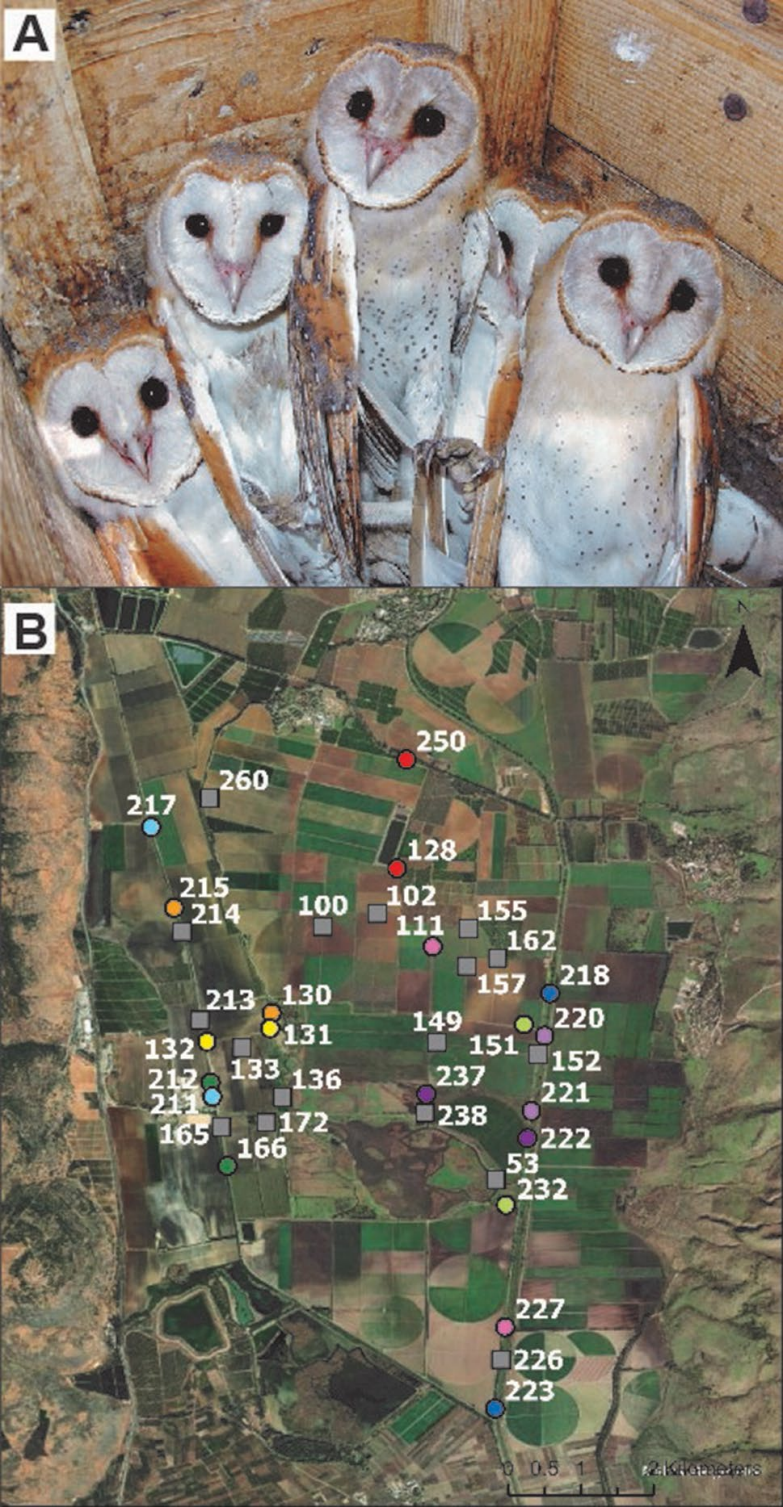
(**A**) Barn owl nestlings from one of the monitored nest boxes. (**B**) Location of the nest boxes in the Hula Valley, Israel. Nest boxes that were paired with each other for the exchange of nestlings are depicted with circles of the same color. Nest boxes that were not involved in the cross-fostering experiment (unmanipulated nests) are marked with gray squares

We studied barn owls in northern Israel (Fig. 1) where nest boxes in the Hula Valley were monitored from April 12 - July 5 in 2017 by one of the authors (M.C.) to collect reproductive data [48, 56]. Adults were sampled from both unmanipulated and cross-fostered nests. In many cases, both adults attending the nest were captured at least once to sample their microbiota (see Supplemental Methods), but it was easier to capture the females since they remained in the nest more than the males. As a result, not all males were captured, and some females were captured multiple times (see below for sample sizes).

A partial cross-fostering experiment was conducted between ten pairs of nest boxes (Fig. 1). Nest boxes were paired if they contained a similar number of nestlings of the same age (i.e. equal numbers or only one nestling different). The microbiota of the nestlings was sampled at two developmental time points, one before they began to develop adult feathers at ~ 35 days [55] and a second time before they fledged and began to leave the nest, which in Israel typically occurs after nestlings are 60 days old. When the nestlings were 26–34 (mean = 30.27, standard deviation = 2.56) days of age (which we subsequently refer to as “young” nestlings) they were swabbed to collect a sample of their microbiota (details below and in Supplemental Methods), and then offspring were reciprocally exchanged between the paired nests. Thereafter, the nests contained some nestlings housed with their biological parents (controls) and some nestlings moved from another nest (cross-fostered). Fourteen nests cross-fostered two nestlings and five nests cross-fostered one nestling. One nest (nest 222) had two control nestlings and one cross-fostered nestling from its paired nest (nest 237), but did not donate any cross-fostered nestlings to nest 237. The lack of a cross-fostered nestling in nest 237 did not affect our analyses because none of the nestlings in nest 237 survived to be sampled when they were older. There were no significant differences (*F*_1,61_ = 0.75, *P* = 0.391) in nestling age between the control (mean = 30.0 days, min = 26, max = 34, standard deviation = 2.56) and cross-fostered nestlings (mean = 30.6 days, min = 26, max = 34, standard deviation = 2.58) at the start of the experiment, nor at the second sampling time (*F*_1,42_ = 0.74, *P* = 0.395). The average brood size of the nests in the experiment was 5.35 nestlings (standard deviation = 1.75, min = 3, max = 8) and in this population there were an average of 5.35 fledglings per pair (*N* = 220, min = 1, max = 11) [57].

The nestlings were swabbed again for microbiota profiling typically around three weeks after the exchange between the nest boxes (median = 22 days, min = 18 days, max = 28 days), when the nestlings were 49–56 days (mean = 53.11, standard deviation = 1.81) of age (which we subsequently refer to as “old” nestlings). In addition to the 20 nest boxes involved in the experiment, microbiota samples were also collected from nestlings in 18 unmanipulated nest boxes (Fig. 1) when their nestlings were 46–56 days of age (unmanipulated nestlings were only sampled at the old time point). In addition, twelve nestlings from eight experimental nests were only swabbed at the second time point, because they were a priori excluded from the cross-fostering experiment (we refer to them as “excluded” nestlings). These nestlings all remained with their biological parents (like designated control nestlings) but were excluded from the experiment because we wanted approximately balanced sample sizes of control and cross-fostered offspring that were of similar ages. The excluded nestlings were younger than the nestlings designated as controls in their nest. Although excluded nestlings were not used in the analyses of the cross-fostering experiment, they were used in other analyses of the microbiota such as comparisons testing for age differences, sex differences, and the influence of the parents. Sample sizes for each type of nestling are provided below.

We sampled the cloacal and oral microbiota (see Supplemental Methods) to study the effects of cross-fostering on two different bacterial communities. Cloacal swabs were collected from all nestlings and adults. Oral swabs were collected only from young and old nestlings in the cross-fostering experiment to provide data on an additional microbial community that could be examined as part of our experiment. The mass (g) of each nestling and adult was measured each time it was swabbed. We were able to determine the sex of all the adults and most of the nestlings (sample sizes below) using the methods described in the Supplemental Methods.

### Movement data

We collected data on the movement behavior of adult owls using a reverse-GPS ATLAS tracking system [58, 59]. Tags were attached to adult owls using a Teflon backpack harness. Owls could be localized as often as once every four seconds, but often the frequency of localization was less than the maximum. A variety of factors could temporarily obscure the signal of an ATLAS tag including the owl being in a place of poor coverage due to landscape features, the owl dropping to ground level when hunting, and the signals being jammed by communication disturbances. However, the resulting data can still include tens of thousands of localization records [48]. Only data pertaining to nighttime (an hour before sunset until an hour after sunrise) were included in our analysis, because barn owls in Israel are strictly nocturnal and remain entirely inactive during daylight hours, initiating hunting activity only post-sunset and returning to their roosts prior to sunrise. A speed and median filter were implemented on the data to eliminate/correct any erroneous points [60]. We summarized the movement data during a 13-day window that began the day after the capture of the adult owl to prevent any effect of capture on subsequent movement behavior from impacting the results. We set a minimum threshold level of data per day by excluding any days with less than 20% of the expected number of localizations. The average number of effective days of data was 8 days, with a range from 2 to 13 days.

Owls may spend significant amounts of time near the nest, performing important tasks such as monitoring for predators and feeding the nestlings or waiting to feed them. The amount of time in attendance near the nest could increase opportunities for direct microbial exchange through behaviors like feeding and grooming, as well as indirect exchange such as the transfer of microbes into/from the local environment. We measured the percentage of time the owl was at the nest box to quantify the amount of time the adult owl was in close proximity to the nestlings. The percentage of time at the nest box was calculated by dividing the number of observations in which the owl was within a 100 m radius of the nest by the total number of observations. Movement data were obtained for 10 mothers and 9 fathers from experimental nests and 14 mothers and 6 fathers from unmanipulated nests. We used an arcsin square root transformation of the percentage of time at the nest box to improve the fit of the data to a normal distribution in our statistical tests, but we used the more comprehendible untransformed metric when visualizing the data in the figures.

### DNA extraction and sequencing

Bacteria in DNA extraction kits can lead to confounding batch effects if biological groups of samples are separately processed with different kits [61, 62]. Therefore, swabs were randomly assigned to different sets of samples for DNA extraction together. To identify contaminants, we included nine negative control tubes that did not have a swab added to them, but each tube went through the extraction procedure alongside a set of owl samples. We also included two blank controls, which were UltraPure distilled water (Invitrogen) that did not go through the DNA extraction process but went through the PCR amplification procedures. DNA extraction was performed with Qiagen PowerLyzer PowerSoil DNA Kits (see Supplemental Methods). The V4 region of the 16S rRNA gene was amplified for each sample at the Argonne Sequencing Center with triplicate PCRs (see Supplemental Methods) and then sequenced on a 150 bp paired-end run of an Illumina HiSeq 2500 machine. Most of the PCRs for the adult owls studied previously [48] were sequenced again alongside the nestling samples to achieve a higher sequencing depth. We excluded adult samples that were not resequenced with the nestlings, because exploratory analyses suggested that the earlier MiSeq data was not comparable to the HiSeq data.

### Sequence data processing

The 16S rRNA gene sequence data were processed in R [63] using the pipeline of Callahan et al. [64] to determine the frequency of different amplicon sequence variants (ASVs) [65]. After removing the initial 10 bases of each read, we used DADA2 [66] to infer ASVs from a combined pool of all of the sequence data. Then paired reads were merged, chimeric sequences were filtered out, and the sequences were classified with the SILVA 138.1 taxonomy database [67, 68] obtained from http://benjjneb.github.io/dada2/training.html. The sequences were aligned with DECIPHER [69] in order to infer a maximum likelihood phylogeny in phangorn [70]. We used phyloseq [71] to combine the ASV table, the taxonomic information, the phylogeny, and metadata about the samples for subsequent data analyses. We used decontam [72] with a 0.5 prevalence filter threshold to filter out ASVs that were more prevalent in the negative and blank controls than in the biological samples, which led to 1105 ASVs being removed and 16,361 ASVs being retained. We also excluded ASVs classified as mitochondria or chloroplasts, ASVs not classified to a phylum, and any non-bacterial ASVs. After filtering, the average number of reads across samples was 143,260, with a minimum of 169 and a maximum of 429,644 per individual. The samples with the lowest numbers of reads had low post-PCR concentrations after 16S rRNA gene amplification, which suggested they had sample quality issues. The sample with the lowest number of reads but with good average post-PCR amplification (> 9 ng/µL) had 9912 reads. Thus, we excluded eight samples with less than 9000 reads. To standardize the data [73], we rarified the data (random number seed = 999) so that each sample had the same amount of sequencing data (9912 reads). We then excluded two samples whose identity was uncertain, four duplicates where the same sample was run twice, and all samples (16 in total) with poor post-PCR concentrations (< 9 ng/µL), which tended to have abnormally low alpha diversity and are considered failed libraries by Argonne National Labs.

Our final dataset had the following sample sizes. For cloacal swabs from adults, we had 72 female samples from 54 unique individuals and 26 unique male samples (males were only sampled once). We filtered the data so that only a single sample per adult female was used, keeping the sample taken at the closest time to when the old nestlings were sampled, to avoid having duplicate adult female samples in our analyses. For the comparisons of old nestlings to their parents, we had data for 12 experimental fathers, 15 experimental mothers, 18 unmanipulated mothers, and 8 unmanipulated fathers. For cloacal swabs from nestlings, we had 85 females, 62 males, and 28 individuals of unknown sex. For the nestling cloacal swab samples, there were 61 control (36 young, 25 old), 46 cross-foster (27 young, 19 old), 12 excluded (old, see above), and 56 unmanipulated (old). For oral swabs from nestlings, there were 60 control (39 young, 21 old) and 45 cross-foster (30 young, 15 old) samples, which included 47 females, 32 males, and 26 individuals of unknown sex.

### Statistical tests

We used R 4.0.4 [63] for our statistical tests and visualized the data with the R-packages ggplot2 [74], ggthemes [75], and gridExtra [76]. We used the typical significance threshold of α < 0.05 for our tests. Four of our hypotheses were directional (i.e. Increased Alpha Diversity, Increased Microbiota Change, Higher Similarity to Mothers than Fathers, Reduced Similarity to Parents with Cross-fostering), which would justify the use of one-tailed tests. However, we used the more conservative two-tailed tests for these hypotheses to help offset any increased risk of false positives that came from repeating our tests across different metrics of the microbiota (see below). We used the plot function of lmne to examine the fitted values versus the standardized residuals to look for heteroscedasticity and the qqnorm function to assess the normality of the standardized residuals. The only issue with the residuals that we detected was caused by an extreme outlier identified in the oral swab data, which was dealt with by analyzing the data with and without the inclusion of the outlier. Extreme outliers were identified with the R-package rstatix [77] using boxplot methods.

We measured the alpha diversity of the samples using two metrics: the observed number of ASVs (which we refer to as “observed diversity”) and the Chao1 estimator of the number of species [78, 79], which in our case estimates the total number of ASVs of the bacterial community (i.e., observed ASVs plus an estimate of ASVs missed in the sample). Both metrics were log_10_ transformed and the transformed data sufficiently approximated a normal distribution as assessed with histograms and qqplots. The observed and Chao1 metrics were highly correlated (e.g. for young nestling cloacal swabs *R*^*2*^ = 0.85, old nestling cloacal swabs *R*^*2*^ = 0.86, both *P* < 1 × 10^− 15^) so we only report the tests with log_10_ observed ASV diversity (hereafter solely referred to as observed diversity) unless there was a discrepancy between the two metrics, in which case some caution is warranted because the results are dependent upon the particular assumptions behind how the metrics are calculated. To test whether the following factors were associated with alpha diversity, we used linear mixed-effects models implemented in nlme [80] and summarized with the Anova function from the car package [81] with mass as a covariate to account for the development of the owls and random intercepts for the natal nest box to control for the shared natal environment and genetics of related nestlings. We tested whether alpha diversity differed between experimental treatments (i.e., control and cross-fostered nestlings) to test the Increased Alpha Diversity Hypothesis. We tested the Increased Microbiota Change Hypothesis by determining whether the change in alpha diversity (old minus young alpha diversity) was associated with experimental treatment using nestlings that had data from both the young and old time points and with the change in mass as a covariate. We also tested whether the alpha diversity of the mother, alpha diversity of the father, percent of time the mother was at the nest box, and percent of time the father was at the nest box were related to nestling alpha diversity at the old time point (i.e. testing the Higher Similarity to Mothers Hypothesis and Parental Movement Ecology Hypothesis). We tested whether male and female nestlings had different alpha diversity (Sexual Differentiation Hypothesis) with a dataset that excluded nestlings with undetermined sex. We tested whether the alpha diversity of young nestlings, old nestlings, and adults differed from one another (Developmental Changes Hypothesis). For nestlings with data from both time points, we tested whether there was a correlation between their young and old alpha diversity, with their mass when old as a covariate (Developmental Changes Hypothesis).

We quantified the differences and changes in bacterial community compositions (i.e., beta diversity) in phyloseq using the Jaccard, Bray-Curtis, unweighted UniFrac, and weighted UniFrac metrics. These metrics vary in how they quantify dissimilarities in ecological communities, thereby allowing us to explore whether our results changed with methodology and what aspects of the bacterial communities (i.e. presence/absence vs. abundance of taxa) differed among groups [82]. Jaccard and unweighted UniFrac consider only the presence/absence of ASVs whereas Bray-Curtis and weighted UniFrac also utilize information on the abundance of the ASVs (i.e. how many reads are assigned to each ASV, which provides data about how common an ASV is in a sample). Unweighted and weighted UniFrac additionally incorporate phylogenetic distance between ASVs when calculating distances [82, 83]. We used Jaccard as the primary metric in the paper, but report the other metrics to identify situations in which: (1) all methods are concordant and the results are robust to choice of methodology, (2) Jaccard and unweighted UniFrac show similar results, but differ from Bray-Curtis and weighted UniFrac, which means that ASV abundance information changes the patterns, and (3) only a single metric shows a significant pattern and the results therefore depend on the particular assumptions of that metric. Conducting the same test with four different metrics increases the risk of a false positive due to the multiple tests. Standard approaches for adjusting *P*-values for multiple testing, like the Dunn-Šidák method, assume that the tests are independent from one another [84], but our multiple tests are not independent from one other, because the four metrics utilize the same underlying data and thus can be correlated. Therefore, we report the *P-*values without a multiple testing correction, but interpret *P-*values close to 0.05 with caution and not as definitive support. We quantified dissimilarities in bacterial community compositions between groups of interest (e.g. paired nest boxes, males and females, age groups), changes in the microbiota over time within an individual (young vs. old time points for a nestling), and differences between the microbiota of a nestling and its mother or father. We used a linear mixed-effects model in nlme to test whether the degree of dissimilarity in beta diversity between the young and old time points for an individual was correlated with the experimental treatment along with random intercepts for the natal nest to test the Increased Microbiota Change Hypothesis. We conducted similar models with random intercepts for the natal nest to test if the microbial community dissimilarity between nestlings and their parents: (1) differed for their mother and father (Higher Similarity to Mothers Hypothesis), (2) was related to the percent of time a parent was at the nest (Parental Movement Ecology Hypothesis), and (3) differed between control and cross-fostering nestlings (Reduced Similarity to Parents with Cross-fostering Hypothesis). We did not consider the Bray-Curtis metric for these three tests, because the Bray-Curtis distances between parents and offspring were not normally distributed and could not be transformed to match a normal distribution.

We tested whether groups of samples differed from one other with a permutational multivariate analysis of variance (PERMANOVA) using 9999 permutations, which was implemented with the adonis function in the R-package vegan [85]. In conjunction with these tests, we tested for homogeneity of group dispersions using the betadisper function in vegan, because PERMANOVA tests can be sensitive to both position of the group centroids as well as differences in group dispersion when the groups have unbalanced sample sizes [86]. We used these tests to evaluate whether the microbial communities of young nestlings differed from old nestlings, young nestlings differed from adults, and old nestlings differed from adults (Developmental Changes Hypothesis) as measured by the four different ecological community distance metrics. We similarly tested for differences between males and females (Sexual Differentiation Hypothesis) in separate tests for young nestlings, old nestlings, and adults, but with individuals of unknown sex excluded. We also hypothesized that nestlings would have bacterial communities differentiated by their natal nest environment before the cross-fostering experiment, but differences between the paired nests would decrease after cross-fostering (Convergent Bacterial Community Compositions Hypothesis). To test this, we: (1) subset the data to a single pair of nests between which nestlings had been exchanged, (2) tested whether the microbiota of young nestlings exhibited differences between the two nests before the experiment using Jaccard dissimilarity and PERMANOVA, and (3) tested whether the microbiota of old nestlings were differentiated by their natal nest or by the nest they lived in after cross-fostering using Jaccard dissimilarity and PERMANOVA. We similarly tested whether the microbiota of nestlings in pairs of unmanipulated nests were distinct from one another to evaluate whether old nestlings are likely to show nest box differences when no exchanges between nests are made. If cross-fostering leads to a convergence in the microbiota of the nestlings, then we would expect that old nestlings in the experiment would show little differentiation by nest, but that old unmanipulated nestlings would be differentiated by their nests because they did not have an exchange of nestlings. Alternatively, if we do not observe differentiation by nest in both unmanipulated and experimental nests, this would suggest that any convergence observed among old nestlings in the experiment may be due to age effects, but not necessarily bacterial exchange facilitated by cross-fostering. To accomplish the analysis of unmanipulated nestlings, we paired unmanipulated nest boxes that had similar brood sizes (i.e. differing by at most one nestling) when the offspring were 53 days old.

We used all nestling samples (i.e., cross-fostered, control, excluded, and unmanipulated nestlings) for comparisons between the sexes and different age classes and for tests comparing parents to offspring, but focused on just the control and cross-fostered nestlings when looking for effects of the experiment. We restricted the tests comparing offspring to parents to just the nestlings at the old time point, because this allowed us to substantially increase our sample sizes by analyzing both unmanipulated and experimental nestlings. The old nestlings were swabbed on average 29 days (min. = 16, max = 51) after their mother was swabbed and on average 24 days (min. = 14, max = 84) after their father was swabbed. Thus, all nestling samples post-date the adult samples and thus could reflect the exposure to the adult microbiota from an earlier date.

We visualized the differences between the cloacal and oral swab samples via a multidimensional scaling plot of Jaccard distances among the samples and via barplots of the top four most abundant bacterial phyla in the samples. Subsequent analyses were performed separately for the oral and cloacal swab samples, which we found had different microbial communities (see below). We were able to conduct more extensive analyses with the cloacal microbiota data, because oral swabs were limited to just experimental nestlings whereas cloacal swabs were collected for experimental nestlings, unmanipulated nestlings, and adults.

## Results

### Oral and cloacal swab overview

The nestling oral and cloacal environments had highly distinct bacterial communities, with Jaccard distances distinguishing nearly all oral and cloacal samples from one another (Supplemental Fig. 1). The bacterial phyla *Actinobacteroita*, *Bacteroidota*, *Firmicutes*, and *Proteobacteria* were abundant in both cloacal and oral communities (Supplemental Fig. 2). However, barplots of the proportions of these phyla within each sample show that adult cloacal swabs had a high proportion of *Actinobacteroita* (Supplemental Fig. 2A), nestling cloacal swabs had a high proportion of *Firmicutes* (Supplemental Fig. 2B), and nestling oral swabs had a high proportion of *Proteobacteria* (Supplemental Fig. 2C).

### Testing the developmental changes hypothesis

**Fig. 2:**
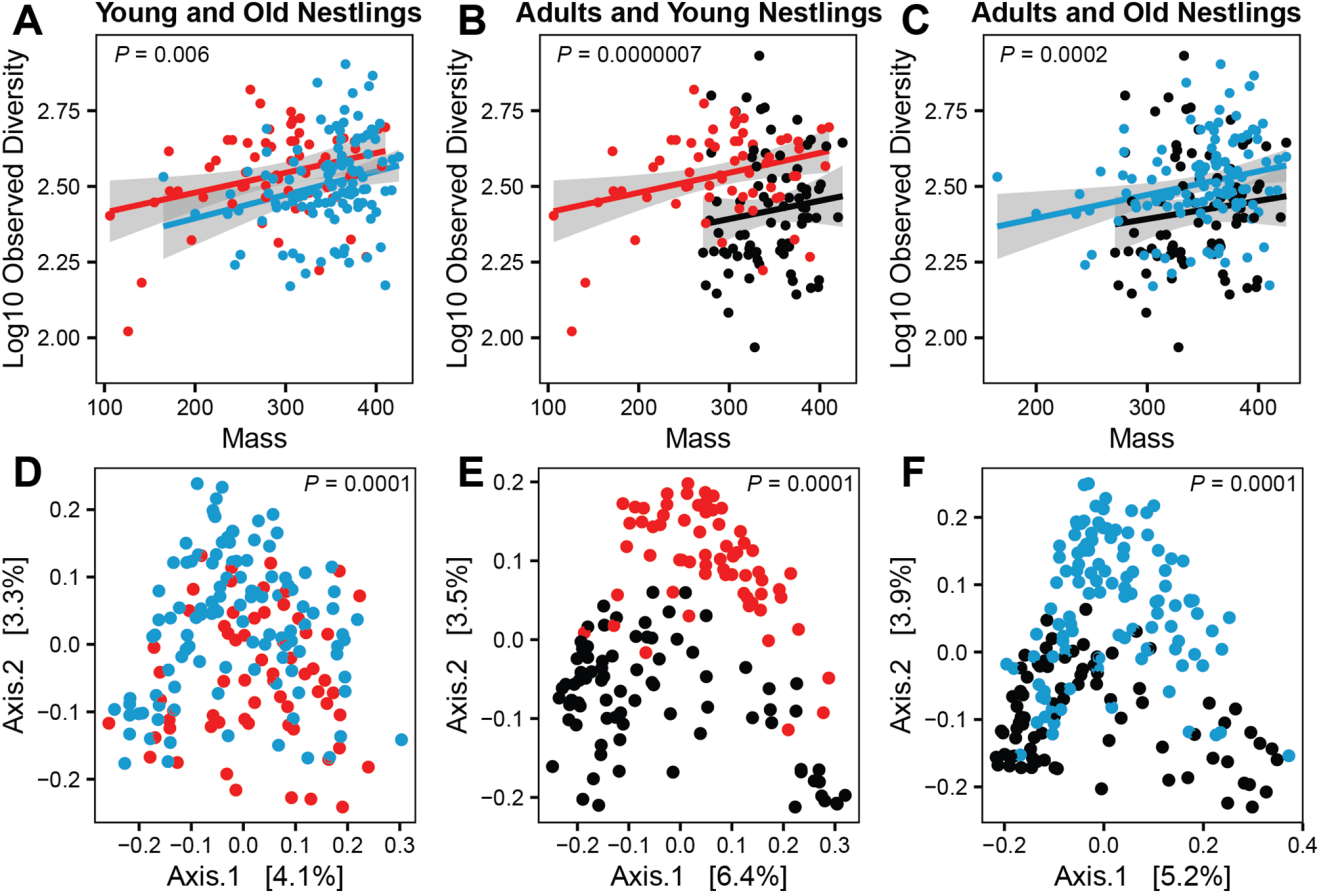
Effects of age on the cloacal microbiota. (**A**–**C**): Alpha diversity in relation to both the mass (g) of the individual and its age class. Linear trend lines along with 95% confidence intervals in gray are depicted for each age group. (**D**–**F**): Changes in bacterial community composition with age as measured by Jaccard dissimilarity and plotted with multidimensional scaling with the percentage of variation explained by each axis given in brackets. Young nestlings = red points, old nestlings = blue points, and adults = black points. *P*-values within the plots are for the effect of age from linear mixed-effect models (panels **A**–**C**) and PERMANOVA tests (panels **D**–**F**)

Younger nestlings had higher observed bacterial diversity than older nestlings for cloacal samples (Fig. 2A, *N*_observations_ = 175, *N*_nests_ = 38, *X*^*2*^_1_ = 7.63, *P* = 0.006) and the mass of the nestling was positively correlated with observed diversity (*X*^*2*^_1_ = 12.43, *P* = 0.0004) in the model. There was no correlation between the observed bacterial diversity at young and old time points for nestlings that had cloacal data at both sampling times (*N*_observations_ = 35, *N*_nests_ = 15, *X*^*2*^_1_ = 0.41, *P* = 0.52; Mass when old: *P* = 0.87). Nestlings had higher cloacal observed diversity than adult owls at both young (Fig. 2B, *N*_observations_ = 141, *N*_nests_ = 54, *X*^*2*^_1_ = 24.59, *P* = 0.0000007; mass *P* = 0.025) and old (Fig. 2C, *N*_observations_ = 190, *N*_nests_ = 55, *X*^*2*^_1_ = 13.70, *P* = 0.0002; mass *P* = 0.009) time points. Younger nestlings had higher observed bacterial diversity for oral swabs (Supplemental Fig. 3A, *N*_observations_ = 105, *N*_nests_ = 20, *X*^*2*^_1_ = 6.07, *P* = 0.014; mass *P* = 0.61). This correlation with age group was stronger (*N*_observations_ = 104, *N*_nests_ = 20, *X*^*2*^_1_ = 14.79, *P* = 0.00012) if a single extreme outlier was removed, which had the highest observed diversity of any other sample. There was no correlation between the observed diversity at young and old time points for all nestlings that had oral data at both sampling times (*N*_observations_ = 32, *N*_nests_ = 13, *X*^*2*^_1_ = 1.88, *P* = 0.17; Mass when old: *P* = 0.62), but there was a significant correlation if the previously identified extreme outlier was excluded (Supplemental Fig. 2B, *N*_observations_ = 31, *N*_nests_ = 12, *X*^*2*^_1_ = 5.69, *P* = 0.017; Mass when old: *P* = 0.010).

Young and old nestlings had significantly different cloacal bacterial community compositions for Jaccard (Fig. 2D, *F*_1,173_ = 2.59, *P* = 0.0001; dispersion *P* = 0.001) and the three other metrics of bacterial community composition (i.e. Bray-Curtis, unweighted UniFrac, and weighted UniFrac; all *P* = 0.0001). Although there were significant differences in dispersion for all comparisons except weighted UniFrac (*P* = 0.14), young and old nestlings were often separated by Axis 2 of a multidimensional scaling plot of their bacterial communities (Fig. 2D), which suggests that they differ in more than just dispersion. Young and old nestlings also differed in their oral bacterial community compositions, which were significantly different for Jaccard (Supplemental Fig. 3C: *F*_1,103_ = 2.40, *P* = 0.0001; dispersion *P* = 0.17) and the three other metrics of bacterial community composition (all *P* < 0.001). The cloacal bacterial community compositions of adults and young nestlings were significantly different for Jaccard (Fig. 2E, *F*_1,141_ = 4.49, *P* = 0.0001; dispersion *P* = 0.001) and the three other metrics of bacterial community composition (all *P* = 0.0001). Adults and old nestlings also had significantly different cloacal bacterial communities for Jaccard (Fig. 2F, *F*_1,191_ = 4.89, *P* = 0.0001; dispersion *P* = 0.21) and the three other metrics of bacterial community composition (all *P* = 0.0001).

### Testing the sexual differentiation hypothesis

Male and female nestlings did not differ in cloacal observed diversity either when young (*N*_observations_ = 38, *N*_nests_ = 15, *X*^*2*^_1_ = 2.73, Sex: *P* = 0.098, Mass: *P* = 0.89) or when old (*N*_observations_ = 109, *N*_nests_ = 34, *X*^*2*^_1_ = 0.76, Sex: *P* = 0.38, Mass: *P* = 0.02) and did not differ in oral observed diversity when young (*N*_observations_ = 43, *N*_nests_ = 15, *X*^*2*^_1_ = 0.46, Sex: *P* = 0.50, Mass: *P* = 0.96) or when old (*N*_observations_ = 36, *N*_nests_ = 14, *X*^*2*^_1_ = 1.86, Sex: *P* = 0.17, Mass: *P* = 0.78).

Adult male and female owls had significant differences in their cloacal bacterial community compositions for unweighted UniFrac *(F*_1,78_ = 1.74, *P* = 0.003; dispersion *P* = 0.013) and Jaccard (*F*_1,78_ = 1.41, *P* = 0.013; dispersion *P* = 0.23) distances, but were not different for weighted UniFrac distances (*F*_1,78_ = 1.21, *P* = 0.27; dispersion *P* = 0.27) or for Bray-Curtis dissimilarity (*F*_1,78_ = 1.10, *P* = 0.31; dispersion *P* = 0.74). Therefore, we tested for sex differences in the nestlings using the unweighted UniFrac and Jaccard distances. For both metrics, there were no significant sex differences in the nestling cloacal or oral bacterial communities for either young or old nestlings (young nestling oral swabs with Jaccard had *P* = 0.086; all other *P* > 0.34).

### Testing the increased alpha diversity hypothesis

**Fig. 3:**
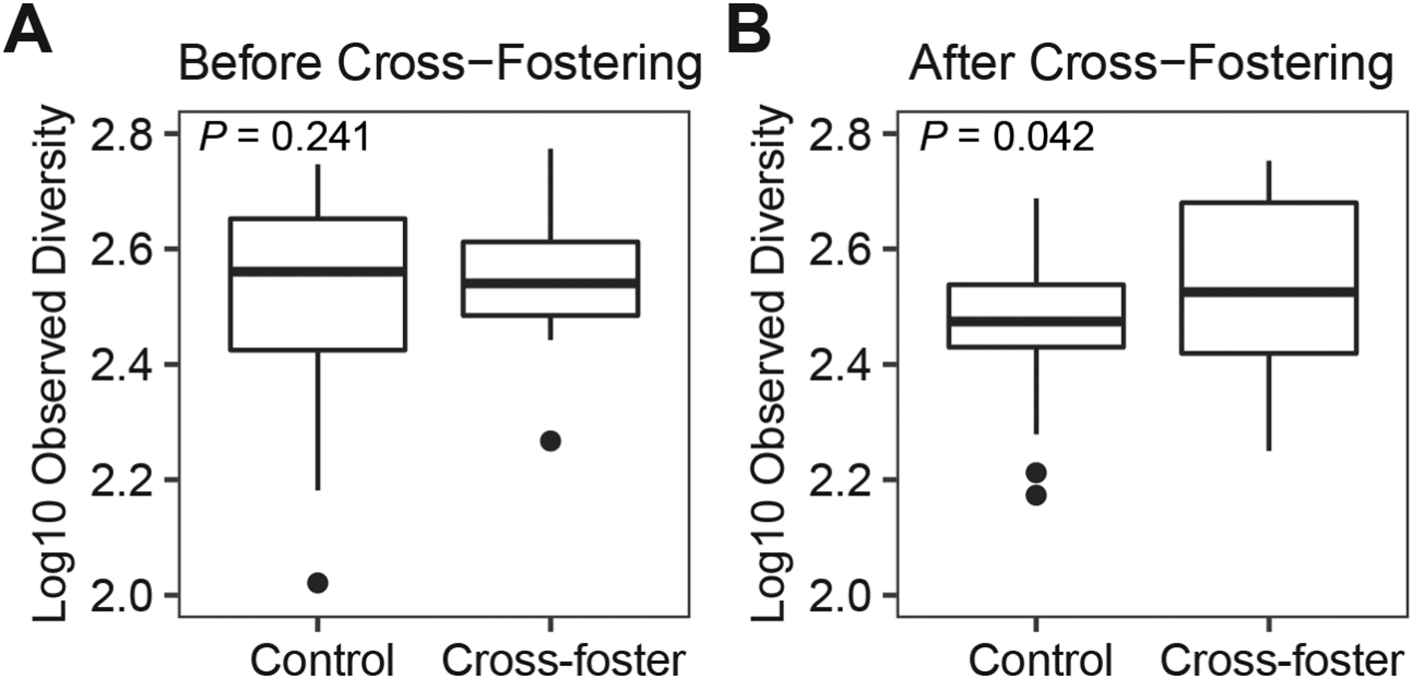
Alpha diversity before and after the cross-fostering experiment. The boxplots show the median as a thick line within boxes of the 25th and 75th percentiles, with whiskers for the minimum and maximum, and outliers as separate points. *P*-values within the plots are for the test of whether control and cross-foster offspring differed from one another. The sample sizes are 36 control and 27 cross-foster nestlings in 20 nests in the Before Cross-Fostering plot (**A**) and there are 25 control and 19 cross-foster nestlings in 16 nests in the After Cross-Fostering plot (**B**)

Before the cross-fostering experiment began, the cloacal observed diversity did not differ between the nestlings assigned to the control and cross-foster groups (Fig. 3A, *N*_observations_ = 63, *N*_nests_ = 20, *X*^*2*^_1_ = 1.37, *P* = 0.24; nestling mass *P* = 0.042). Approximately three weeks after the cross-fostering manipulation, the cross-fostered nestlings had significantly higher observed bacterial diversity than the control offspring (Fig. 3B, *N*_observations_ = 44, *N*_nests_ = 16, *X*^*2*^_1_ = 4.13, Experiment: *P* = 0.042, Mass: *P* = 0.26), but this relationship was not significant when measured by Chao1 (Experiment: *P* = 0.099, Mass: *P* = 0.26). After cross-fostering, the control offspring had on average 306 ASVs, whereas the cross-fostered offspring had an average of 375 ASVs. For oral swabs, there was no significant difference in observed diversity between the control and cross-fostered old nestlings (*N*_observations_ = 36, *N*_nests_ = 14, *X*^*2*^_1_ = 1.46, Experiment: *P* = 0.23, Mass: *P* = 0.71).

### Testing the increased microbiota change hypothesis

**Fig. 4:**
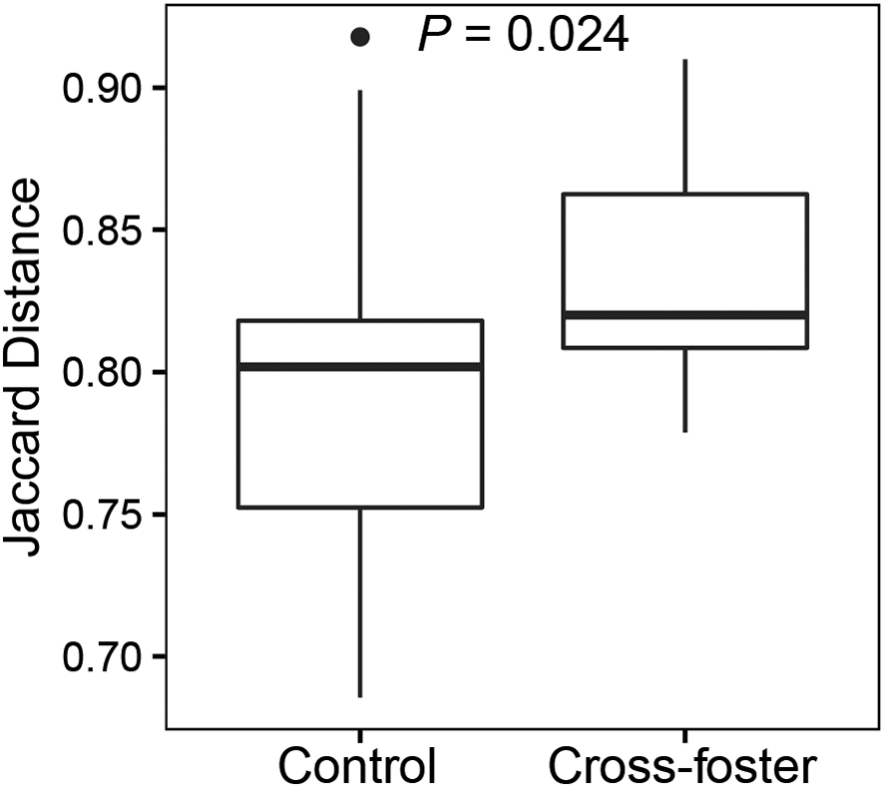
Change in the microbial community composition in relation to the cross-fostering experiment. The amount of change in the microbiota for each individual nestling when it was young vs. old was measured by the Jaccard metric and then summarized across individuals with boxplots. The *P*-value is for the test of whether control and cross-foster offspring differed from one another. This test had 20 controls and 15 cross-fostered nestlings. The boxplots show the median as a thick line within boxes of the 25th and 75th percentiles, with whiskers for the minimum and maximum, and the outlier as a separate point

The amount of change in observed diversity (i.e. alpha diversity) between old and young time points was not influenced by experimental treatment (*N*_observations_ = 35, *N*_nests_ = 15, *X*^*2*^_1_ = 1.33, Experiment: *P* = 0.25, Mass Change: *P* = 0.31). However, cross-fostered nestlings had greater changes in their cloacal microbial community compositions (i.e. beta diversity) between the young and old time points than control nestlings (Fig. 4, Supplemental Fig. 4), which was significant for Jaccard (*N*_observations_ = 35, *N*_nests_ = 15, *X*^*2*^_1_ = 5.08, *P* = 0.024), Bray-Curtis (*X*^*2*^_1_ = 4.21, *P* = 0.040), and weighted-UniFrac (*X*^*2*^_1_ = 6.87, *P* = 0.009), with a similar, but not significant, trend for unweighted UniFrac (*X*^*2*^_1_ = 3.54, *P* = 0.060). Control and cross-fostered nestlings did not significantly differ in changes to their oral bacterial communities between young and old time points for Jaccard (*N*_observations_ = 32, *N*_nests_ = 13, *X*^*2*^_1_ = 0.14, *P* = 0.71) and the other three metrics of bacterial community composition (all *P* > 0.2).

### Testing the convergent bacterial community compositions hypothesis

**Fig. 5:**
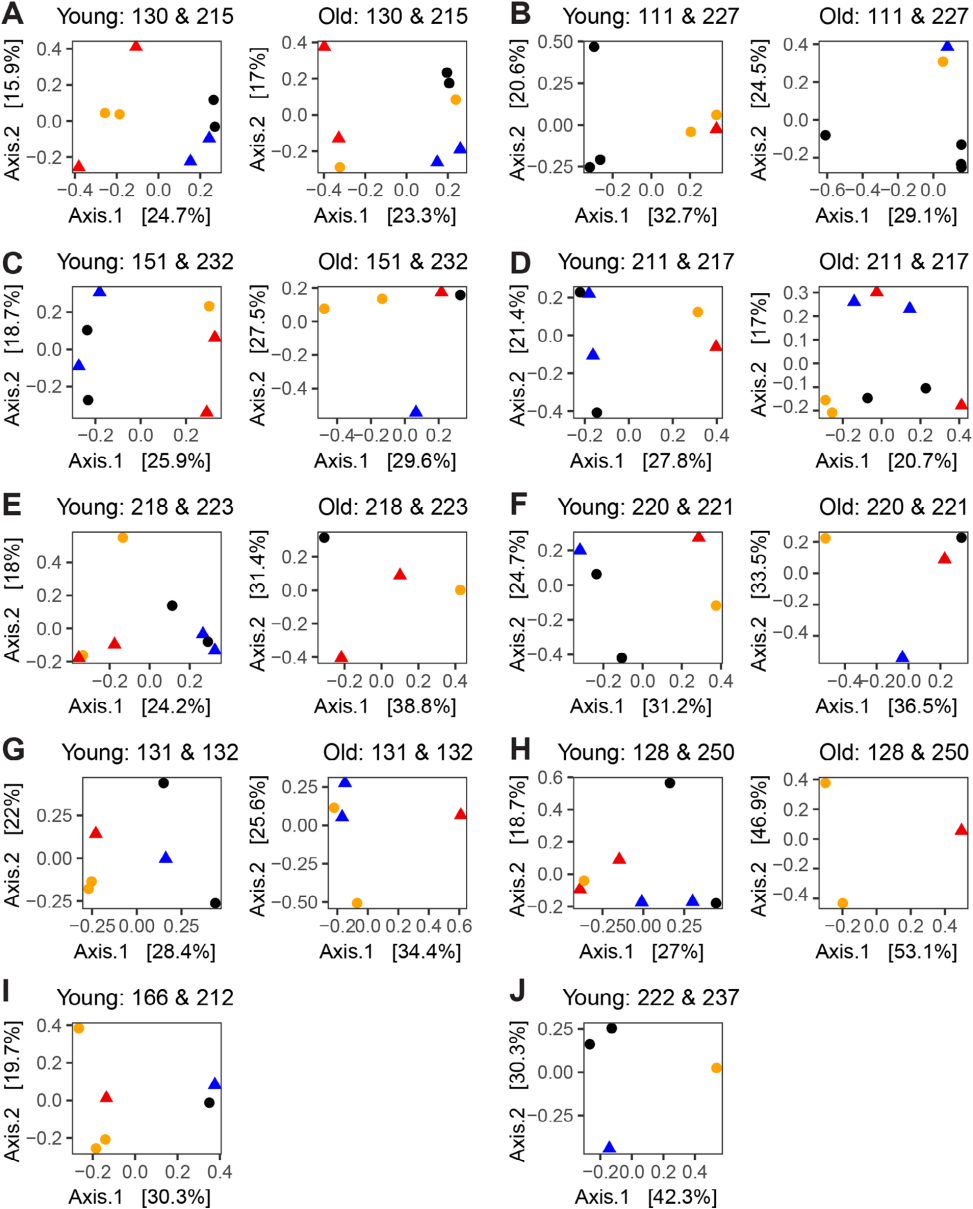
The microbial community composition of the offspring within nests that were paired in the cross-fostering experiment. Each pair of nests (numbers above graphs) is shown at the initial time point before the cross-fostering experiment when the nestlings were young (left) and after cross-fostering when the nestlings were ~ 3 weeks older (right). Nestlings are denoted by their initial nest and experimental category, with Nest 1 control = orange circles, Nest 1 cross-fostered = red triangles, Nest 2 control = black circles, Nest 2 cross-fostered = blue triangles. The number of points between the plots for young and old nestlings typically differs because of nestling mortality, but sometimes points are missing due to lack of data for an individual at a particular time point. No plots are given for old nestlings in plots I and J, because of nestling mortality. The Jaccard dissimilarity for each pair of nests at each time point was separately calculated and plotted with multidimensional scaling, with the percentage of variation explained by each axis given in brackets

We used Jaccard distance to further explore how the cloacal bacterial communities changed in the nestlings from each separate pair of nests in the cross-fostering experiment. We tested whether the pairs of experimental nests had distinct microbial communities from one another before and after the cross-fostering experiment. Before the experiment, the nestlings’ microbiota was differentiated by their nest of origin, because there was no overlap between nestlings from different nests in multi-dimensional scaling plots of their microbial communities (Fig. 5, plots of young nestlings). Although there were clear differences between the pairs of nests before the experiment (Fig. 5), the power of the PERMANOVA tests to evaluate these differences appeared to depend on the sample size of nestlings in the test (i.e. the total number of nestlings in the pair of nests). The differences between nests were significant for the three pairs of nests with the largest sample sizes of offspring (Fig. 5A, C, E; Table 1; nests 130 & 215: *N* = 8, *P* = 0.030, nests 151 & 232: *N* = 7, *P* = 0.029, nests 218 & 223: *N* = 8, *P* = 0.031). Nest pairs with a sample size of at least five nestlings had *P*-values ≤ 0.10 (Fig. 5B, D, F-I; Table 1; nests 128 & 250: *N* = 7, *P* = 0.057, nests 166 & 212: *N* = 6, *P* = 0.067, and the other three nest pairs had *P* = 0.100), and a nest pair with only four nestlings had *P* = 0.25 (Fig. 5J, nests 222 & 237). In contrast, after the experiment no significant microbial community differences were detected among nestlings grouped either by their natal nest box or by the nest box where nestlings lived after the cross-fostering experiment for any of the nest pairs, even for pairs of nests with the largest (*N* = 8) sample sizes (Fig. 5, plots of old nestlings; Table 1). Often the nests were no longer distinct because of the grouping of cross-fostered offspring with the nestlings of their new nest (Fig. 5B, C, F, G), which is the expected pattern of change if cross-fostered offspring have bacterial communities that converge towards their new nest environment. However, there was a case of a control offspring grouping with the offspring of the other nest (Fig. 5A), a pattern which could arise from bacterial transfer from a cross-fostered nestling to a non-related control nestling in its adopted nest. Determining the direction of change of the microbial communities is difficult in the remaining nests due to complex patterns of change (Fig. 5D, but note some similarity of control offspring) or lack of sufficient numbers of surviving offspring to discern the effects of the experiment (Fig. 5E, H, I, J).

**Table 1:** Tests of whether the nests that were paired in the cross-fostering experiment had nestlings with different microbial community compositions. PERMANOVA tests of Jaccard dissimilarities among nestlings were performed to evaluate differences among the nest boxes (i.e., box *F* and *P*-value). The dispersion *P-*value comes from a test of homogeneity of group dispersions. Independent sets of tests were performed for young nestlings and old nestlings. Old nestlings were tested to determine whether they were differentiated by either their natal nest box (1st nest box) or by the nest box where they were raised during the cross-fostering experiment (2nd nest box). The sample size (N) of nestlings in the test is the sum of all the nestlings in the pair of nests. The two tests with the old nestlings had the same sample sizes

Nest groups	Young *N*	Young nestlings 1st box			Old *N*	Old nestlings 1st box			Old nestlings 2nd box		
		Box F	Box *P*	Dispersion *P*		Box F	Box *P*	Dispersion *P*	Box F	Box *P*	Dispersion *P*
130 & 215	8	1.86	0.030	0.536	8	1.24	0.142	0.025	1.12	0.285	0.978
111 & 227	6	1.91	0.100	0.401	6	0.98	0.500	0.001	1.29	0.067	0.268
151 & 232	7	1.73	0.029	0.722	5	1.05	0.400	0.308	1.17	0.200	0.008
211 & 217	6	1.53	0.067	0.001	8	1.02	0.464	0.387	1.22	0.116	0.934
218 & 223	8	1.79	0.031	0.538	4	1.03	0.500	0.042	1.14	0.250	0.042
220 & 221	5	1.33	0.100	0.008	4	0.95	1.000	0.042	1.10	0.333	0.042
131 & 132	6	1.51	0.100	0.101	5	0.97	0.700	0.008	1.56	0.200	0.008
128 & 250	7	1.59	0.057	0.348	3	NA	NA	NA	1.13	0.333	0.167
166 & 212	6	1.71	0.067	0.001	0	NA	NA	NA	NA	NA	NA
222 & 237	4	1.44	0.250	0.042	0	NA	NA	NA	NA	NA	NA

The change from distinct microbial communities before cross-fostering to indistinct communities after the cross-fostering could be due to microbial exchange among the nests facilitated by the movement of cross-fostered offspring or by changes to the microbial communities as the nestlings aged. To distinguish between these scenarios, we assessed whether old nestlings in pairs of unmanipulated nests were distinct from one another (i.e. whether there was a significant effect of the nest on the microbial communities). Multi-dimensional scaling plots of the unmanipulated nests (Supplemental Fig. 5) showed that nestlings were highly distinguished by their nest of origin for five pairs of nests (Supplemental Fig. 5A-E), with clustering by the nest of origin also apparent, but less pronounced, in the remaining two pairs of nests (Supplemental Fig. 5F-G). Five pairs of unmanipulated nests had significant differences in bacterial communities from one another (Supplemental Table 1, Supplemental Fig. 5A-E), one pair had a nearly significant difference (*P* = 0.059, Supplemental Fig. 5F), and one pair was not significantly different (*P* = 0.67, Supplemental Fig. 5G), but only had a sample size of four nestlings.

### Testing the higher similarity to mothers than fathers hypothesis

**Fig. 6:**
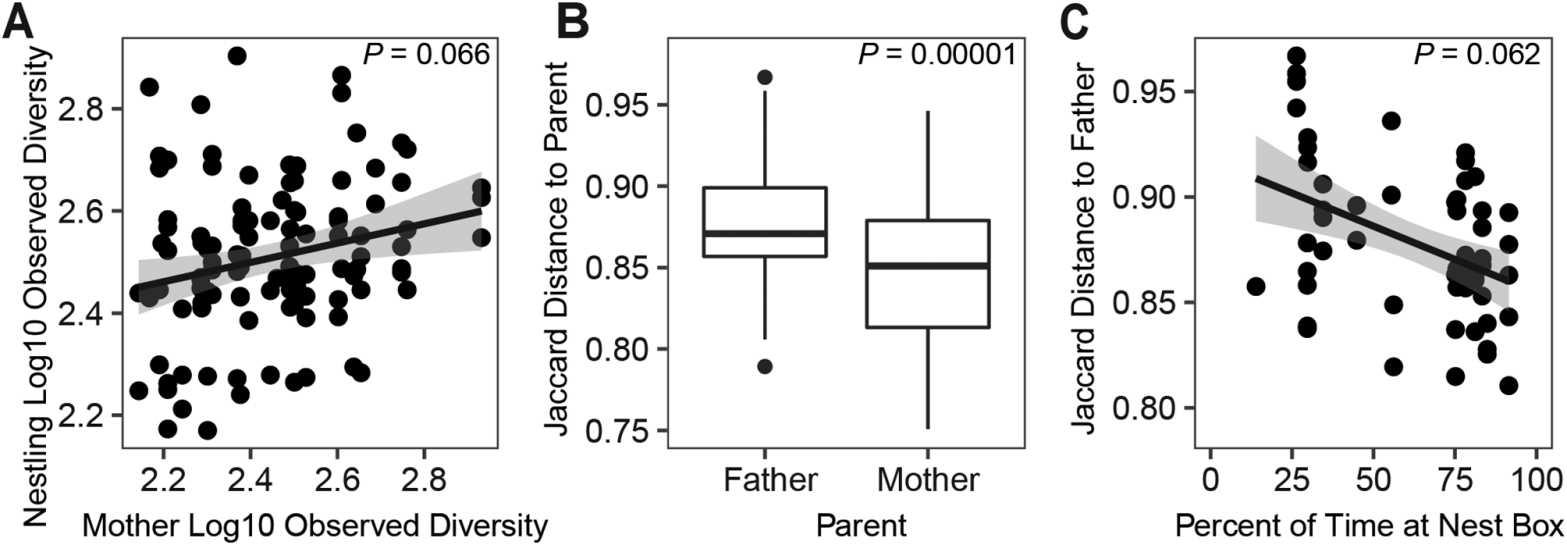
Relationships between the microbiota of the nestlings with their parents. (**A**) The relationship between the observed diversity of the nestlings with observed diversity of their mother. (**B**) The Jaccard distance between nestlings and each of their parents summarized across individuals with boxplots. The *P*-value is from a test of whether nestlings have different distances to their fathers and mothers. (**C**) Relationship between the percent of time an adult male spent at the nest and the Jaccard distance between that male’s microbiota and each of his nestlings. Panels A and B include linear trend lines along with 95% confidence intervals in gray and *P-*values for the correlation. The boxplots in panel C show the median as a thick line within boxes of the 25th and 75th percentiles, with whiskers for the minimum and maximum, and outliers as separate points

We examined whether parents had similar microbiota to their biological offspring using data for all old nestlings. The cloacal observed bacterial diversity of nestlings was not significantly related to the observed diversity of their mother (Fig. 6A, *N*_observations_ = 111, *N*_nests_ = 33, *X*^*2*^_1_ = 3.38, *P* = 0.066; Mass *P* = 0.036), but a borderline significantly positive correlation was observed when Chao1 was used (Supplemental Fig. 6A, *X*^*2*^_1_ = 4.09, *P* = 0.043; Mass *P* = 0.093). The cloacal observed diversity of nestlings was not correlated with the observed diversity of their father (Supplemental Fig. 6B, *N*_observations_ = 69, *N*_nests_ = 20, *X*^*2*^_1_ = 0.02, *P* = 0.885; Mass *P* = 0.21). Comparisons of cloacal bacterial community compositions showed that old nestlings were significantly more distant from their father than their mother for Jaccard (Fig. 6B, *N*_observations_ = 180, *N*_nests_ = 33, *X*^*2*^_1_ = 19.17, *P* = 0.00001), and unweighted UniFrac (*X*^*2*^_1_ = 24.84, *P* = 0.0000006), but were not significantly different with weighted-UniFrac: *X*^*2*^_1_ = 0.83, *P* = 0.36), which incorporates bacterial abundance.

### Testing the reduced similarity to parents with cross-fostering hypothesis

There were no significant effects of the cross-fostering experiment on the Jaccard distance between the old nestlings and their mother (Supplemental Fig. 6E, *N*_observations_ = 43, *N*_nests_ = 15, *X*^*2*^_1_ = 1.55, *P* = 0.21) or their father (Supplemental Fig. 6F, *N*_observations_ = 31, *N*_nests_ = 12, *X*^*2*^_1_ = 0.016, *P* = 0.90) or any of the three other metrics of bacterial community composition (all *P* > 0.22).

### Testing the parental movement ecology affects microbial similarity to offspring hypothesis

The cloacal observed diversity of nestlings was not correlated with either the percent of time spent at the nest by the father (*N*_observations_ = 56, *N*_nests_ = 15, *X*^*2*^_1_ = 0.18, *P* = 0.67; Mass *P* = 0.51) or by the mother (*N*_observations_ = 81, *N*_nests_ = 24, *X*^*2*^_1_ = 0.08, *P* = 0.77; Mass *P* = 0.17). There was a marginally significant negative correlation between the distance of the bacterial communities of old nestlings to their father and the percentage of time the father spent at the nest box for unweighted UniFrac (All data: *N*_observations_ = 56, *N*_nests_ = 15, Supplemental Fig. 6D, *X*^*2*^_1_ = 4.10, *P* = 0.043), the relationship was marginally nonsignificant for Jaccard (Fig. 6C, *X*^*2*^_1_ = 3.48, *P* = 0.062), and no significant relationship was found for weighted UniFrac (*X*^*2*^_1_ = 1.66, *P* = 0.20). This suggests that fathers that spent more time near the nest box may have more similar microbiota to their offspring in terms of the presence/absence of bacterial species, but not in terms of bacterial abundance. In contrast, no correlation was found between the microbiota distance of old nestlings to their mother and the percentage of time the mother spent at the nest box for Jaccard (Supplemental Fig. 6C, *N*_observations_ = 81, *N*_nests_ = 24, *X*^*2*^_1_ = 0.04, *P* = 0.84) or either UniFrac metric (both *P* > 0.36).

## Discussion

Our cross-fostering experiment revealed that microbial communities within barn owl nestlings rapidly change in response to differences in the host environment. Three aspects of the cloacal bacterial communities of the nestlings changed in response to the experiment. First, we found that cross-fostered offspring had higher bacterial diversity than control offspring that remained in their natal nest (Increased Alpha Diversity Hypothesis marginally supported with observed bacterial diversity, Fig. 3). Second, cross-fostered offspring had greater changes in their microbial community composition (beta diversity) than control nestlings (Increased Microbiota Change Hypothesis supported, Fig. 4). However, increased changes in the microbiota were not observed for alpha diversity (Increased Microbiota Change Hypothesis 3 not supported for alpha diversity), which suggests that alpha and beta diversity respond differently to changes in the environment. Third, pairs of nests in the cross-fostering experiment started with the nestlings’ microbiota differentiated by their nest box, but these differences decreased after cross-fostering (Convergent Bacterial Community Compositions Hypothesis supported, Fig. 5), whereas unmanipulated nests maintained their distinctiveness (Supplemental Fig. 5). All these changes occurred over just the three-week period of the cross-fostering experiment. Similarly rapid changes have been observed in a cross-fostering study of great tits (*Parus major*) in which cross-fostered offspring had greater changes in microbial community composition than control nestlings in only seven days [39]. In addition, a study of captive zebra finches (*Taeniopygia guttata*) found that cross-fostered juveniles had greater similarity to their foster relatives than to their biological relatives in only five days [28]. Thus, cross-fostering studies like ours provide experimental evidence demonstrating that bacterial communities are quite dynamic and can quickly respond to new environmental conditions.

The cross-fostering experiment suggests that the social or nest environment or both must play a substantial role in the microbiota of the nestling barn owls. Nest material can house bacteria that are shared with the internal and external microbiota of individuals at the nest [87], so exposure to a new nest could potentially alter the host microbiota. The social environment of nestlings is also likely to be important, because nestlings spend a substantial amount of time in close proximity with one another and their mother, a situation that is conducive to bacterial exchange [17, 40]. Previous studies that have manipulated the social environment by cross-fostering have observed that the microbial communities of cross-fostered offspring can converge in composition toward their host families [34, 39, 43], can maintain the composition of the original nest [47], or can initially converge towards the host family but later diverge in species specific ways [28]. We observed the convergence of the bacterial communities (beta diversity) of cross-fostered offspring with their new family in multiple pairs of nests (Fig. 5B, C, F, G) and this pattern could occur by the cross-fostered nestlings gaining bacteria from their new nest environment or from their adopted siblings or parents. However, when considering the data from all the nests, the exchange of bacteria among nestlings must be part of the explanation for the increased similarity among the old nestlings in our experiment because of the following observations. In studies of the microbiota, the effects of a partial cross-fostering manipulation are not restricted to the offspring moved to a new nest, because bacterial exchange is possible among all nestlings, including from cross-fostered nestlings to the control nestlings in their new nest. We observed two instances (Fig. 5A, D) in which the control offspring from different nests resembled each other more after the experimental manipulation than before the manipulation. The most likely way for this convergence to occur is through microbial exchange among nestlings (e.g. transfer of microbes from a cross-fostered nestling originally from nest 1 to a control offspring in nest 2), because the nest material and adults at the nest did not change for the control offspring.

We observed multiple potential links between the microbiota of nestlings and their parents. The strongest pattern was that the composition of nestling microbial communities (i.e. beta diversity) was more similar to those of their mother than their father when measured by the presence/absence of bacterial ASVs (Higher Similarity to Mothers Hypothesis supported, *P =* 0.00001 Fig. 6B). This pattern likely results from the mother owl remaining at the nest box with the offspring when the offspring are young, whereas the father forages for the family [55]. The mother must influence which bacteria live in the nestlings, but not the abundance of those bacteria, given that this pattern was highly significant for Jaccard and unweighted UniFrac but was not significant for weighted UniFrac. Unlike beta diversity, we only observed suggestive links between the alpha diversity of parents and their offspring. The cloacal Chao1 diversity of nestlings and their mothers was marginally correlated (Supplemental Fig. 6A, *P* = 0.043), but this relationship was nonsignificant for observed bacterial diversity (Fig. 6A, *P* = 0.066) and no significant relationship was found for fathers. We observed that fathers who spent more time at the nest box may have more similar microbiota to their offspring in terms of the presence/absence of ASVs because a marginally significant correlation was found for unweighted UniFrac (*P* = 0.043, Supplemental Fig. 6D) and the Jaccard metric had a similar but nonsignificant *P-*value (*P* = 0.062, Fig. 6C). It may be unlikely for adult traits to have strong effects on their offspring’s microbiota (at least for alpha diversity) given the observation that nestlings have very different microbiota than adults (Fig. 2). Nonetheless, the observation that the microbial communities (i.e. beta diversity) of nestlings are much more similar to their mother than their father provides clear evidence that the proximity of family members can facilitate the convergence of their microbiota, either because of social transmission of bacteria among family members or due to sharing a similar microbial environment. Studies of humans suggest that social transmission may be particularly important, because not only do the microbiota of the people sharing a house come to resemble one another, but the home environment is also affected by the microbiota of the people living there [17]. Close contact amongst individuals can promote microbial exchange, because similarities in microbial communities have been observed in grooming partners in baboons [37], mated kittiwakes (*Rissa tridactyla*) [36], mammalian predators and prey [18], and mice pups with the mother that nursed them [34]. Although we did not find an effect of the cross-fostering experiment on the similarity between the microbial communities of offspring and their biological parents (Reduced Similarity to Parents with Cross-fostering Hypothesis rejected, Supplemental Fig. 6E, F), this does not preclude social transmission from adults to nestlings. Instead, it may mean that more than three weeks is needed to detect any social transmission of bacteria from adults to nestlings or that social transmission from mothers to offspring decreases when the mother resumes hunting and thus spends more time outside of the nest.

We observed a suggestive pattern (unweighted UniFrac: *P* = 0.043; Jaccard: *P* = 0.062) that nestlings had more similar microbial communities to their fathers when the father spent a greater proportion of his time close to the nest box (*Parental Movement Ecology* Hypothesis marginally supported, Fig. 6C, Supplemental Fig. 6D). When a male spends more time at the nest, there could be increased social transmission of bacteria between the father and offspring. Alternatively, fathers that spent substantial time away from the nest may have lower similarity to the offspring because they are more likely to be colonized by bacteria from diverse environments. The lack of a correlation between the mother’s time at the nest and the similarity of her microbiota to her nestlings (Supplemental Fig. 6C) may be due to social transmission of bacteria having already occurred from the mother to offspring (Fig. 6A, B) when the female remained restricted to the nest during the early part of the nestlings’ lives or later when she continued to feed the nestlings. Our observations add to a growing body of literature that has assessed how the movement behavior of a host relates to their microbiota. Differences in microbiota have been linked to host migratory behavior [22–24], social interactions that result in bodily contact [35, 37], and host locomotion activity [88]. Our prior work on adult barn owls found that the bacterial alpha diversity of the host was higher when the owl traveled a greater distance away from the nest [48]. Taken together, these observations suggest that data on host movement patterns can provide insights into the community composition and diversity of the microbiota.

We did not observe differences in the microbiota of male and female nestlings (Sexual Differentiation Hypothesis rejected). Adult owls exhibit sexual differentiation in their microbiota, because the two sexes differ in microbial community composition and males have lower bacterial diversity than females [48]. These patterns may not be observed in nestlings because they harbor distinct microbiota from adults (Fig. 2). The sex differences in the microbiota of adults could arise because of hormonal differences between male and females that only arise later in life [25]. In addition, nestlings of both sexes are likely to have similar behaviors in the nest, but behavioral differences during breeding could affect the environmental exposure of adults to bacteria, because mothers roost inside the nest boxes more frequently than fathers, and males hunt throughout the entire breeding period in contrast to females that predominantly hunt when the nestlings are older. Whatever the cause, the lack of sex differences in the microbiota of nestlings shows that sex differences in the microbiota are not inherent to barn owls and must arise because of later differentiation between males and females. A similar pattern has been observed in barn swallows (*Hirundo rustica*) in which adults exhibited differences in microbial community composition, but nestlings did not exhibit such differences [52].

We observed major shifts in the microbiota related to the developmental stages of the owls (Developmental Changes Hypothesis supported). Young and old nestlings had different bacterial alpha diversity and community composition (beta diversity) compared to each other and to adults (Fig. 2, Supplemental Fig. 2). The differences in community composition were robust across multiple metrics of community dissimilarity and were found in both cloacal and oral microbial communities (Supplemental Fig. 3C). The changes in the microbiota from young to old nestlings were sufficiently large that there was no correlation in cloacal alpha diversity between these two time points. Similarly large shifts in microbiota with development have been observed in other studies of birds. Our observation of higher alpha diversity in nestlings compared to adults (Fig. 2B-C) has also been observed in captive zebra finches (*T. guttata*) and Bengalese finches (*Lonchura striata*), but higher diversity in adults was observed in barn swallows (*H. rustica*), house sparrows (*Passer domesticus*), and ostriches (*Struthio camelus*) [27–30]. Our observation of different microbial communities in nestlings compared to adults (Fig. 2E-F) was also observed in all the bird species listed above. Early development is a period of profound morphological, physiological, and immunological change (barn owls go from hatchlings to flying juveniles in around 60–65 days), all of which could affect the microbiota. The microbiota could also be inherently dynamic during the establishment phase within a host if there are processes of ecological succession as bacteria establish in a new environment [32].

The nestling oral and cloacal bacterial communities were similarly influenced by nestling development, but generally were influenced by different factors. Both the oral and cloacal microbiota showed differences in observed alpha diversity (Fig. 2A, Supplemental Fig. 3A) and community composition (beta diversity) between young and old nestlings (Fig. 2D-F, Supplemental Fig. 3C). However, the observed alpha diversity of the oral microbiota showed a correlation between young and old time points (Supplemental Fig. 3B) and was not correlated with nestling mass, in contrast to the cloacal microbiota. In addition, the cross-fostering experiment did not affect the oral microbiota, whereas multiple effects of cross-fostering were observed on the cloacal microbiota. The oral and cloacal microbiota of the nestlings were quite different (Supplemental Figs. 1 and 2), which could mean that different factors influence their microbial communities. Although the oral and cloacal environments are connected by an alimentary canal, different parts of the gastrointestinal tract often harbor different microbial communities [31, 89, 90].

## Conclusions

Overall, our research shows that microbial communities substantially change during early development. Our cross-fostering experiment demonstrated that social interactions along with differences in the home environment of an individual can affect both the species diversity and composition of the microbiota in a short period of time. Social transmission of bacteria was further supported by our observation that the microbial communities of nestlings were much more similar to those of their mother, with whom they have prolonged close contact, than to their fathers. Thus, some aspects of the microbiota may reflect ongoing social and environmental transmission, at least during the establishment phase of the microbiota. Cross-fostering offers a rare opportunity to experimentally manipulate wild animals to determine the influence of social, genetic, and environmental factors on their microbiota. Researchers should consider gathering microbial data when conducting cross-fostering studies, which would allow them to examine the microbiota as an additional important axis of biological variation that can influence the energy intake [5–7] and health of a host [8–12].

## Electronic Supplementary Material

Below is the link to the electronic supplementary material.

Supplementary Material 1

Supplementary Material 2

## Data Availability

The sequence data generated for the owl microbiota have been deposited in the Sequence Read Archive (BioProject ID: PRJNA578383, Accession numbers in Additional File: “Barn_owl_nestling_paper_SRA_Accession_numbers.xlsx”) along with associated metadata about the owls [91]. The processed sequence data files and the R-scripts used to analyze the data have been made available on Dryad [92].
