# Supplementary material for: Social, environmental, and developmental factors affect the microbiota of barn owls (Tyto alba) in a cross-fostering experiment
Source: Anim Microbiome. 2024 Dec 24;6:77. doi: 10.1186/s42523-024-00365-w (PMC11667991; doi:10.1186/s42523-024-00365-w)
Supplement: Supplementary file 1 — Supplementary Material 1 [file 42523_2024_365_MOESM1_ESM.docx]

**Supplemental Material for:**

S**ocial, environmental, and developmental factors affect the microbiota of barn owls (*Tyto alba*) in a cross-fostering experiment**

**Supplemental Methods**

Owl capture

Captures took place only after at least one nestling had hatched to minimize the risk of nest abandonment. Adult owls were carefully returned to their nest box with the entrance blocked with a pillow attached to a rope, which was then removed from a distance to reduce the disturbance caused to the owls.

Microbiota sampling

Microbiota samples of the owls were collected in the field with sterile Flexible Mini Tip FLOQSwabs (Copan Diagnostics Inc.). Cloacal swabs were collected from all nestlings and adults by moving the full head of the swab in three circular motions inside of the cloaca. Oral swabs were collected from the nestlings in the cross-fostering experiment by placing the swab inside the mouth and moving the full head of the swab in three circular motions. Swabs were placed inside tubes containing 95% ethanol that were stored for 2-4 days at -20˚C in the field before being moved to a -80˚C freezer.

Sex determination

The sex of adults was typically determined when they were trapped by inspecting them for a brood patch for incubation that is only observed in females. To determine the sex of the offspring (and any unknown adults), feather samples were collected in the field, and a standard genetic test on extracted DNA was used to determine the sex (as detailed in Turjeman *et al.* (2020)). The sex was determined for most of the nestlings, but the sex could not be determined for all nestlings due to lack of feather samples or DNA extraction failure for some individuals.

DNA extraction

We used Qiagen PowerLyzer PowerSoil DNA Kits for DNA extraction. To avoid contamination, we flame sterilized our forceps before using them to place swabs into the bead-beating tubes. We heated the tubes for 10 minutes at 65°C as recommended to facilitate cell lysis and the reaction between the lysis buffer and swab material (MO BIO Laboratories Inc 2018). To increase DNA yield, we used a reduced amount of Bead Solution (615 uL) and Solution C1 (50 uL) so that we did not have to leave behind any of the liquid containing the DNA during the transfer stages of the protocol. We used a PowerLyzer homogenizer for bead-beating, which was done at 3500 rpm for 16 cycles of 30 seconds on and 30 seconds off. The extraction of some of the cloacal swabs sometimes failed to yield sufficient DNA, in which case we did a second extraction from the material remaining in the holding tube that had fallen off the swab. To extract this material, we centrifuged the tube to pellet the cloacal material, pipetted off the ethanol, added Bead Solution plus C1 to the tube, and then pipetted the resulting mix into a bead-beating tube. The original and secondary extraction tubes were then combined, except for one sample for which only the secondary extraction was available.

Samples were concentrated to a volume of 40 µL in a Centrivap vacuum centrifuge. Half of the sample was sent to the Argonne Sequencing Center at Argonne National Laboratory, Lemont, IL, USA where triplicate PCRs were performed to amplify the V4 region of the 16S rRNA gene. The PCR primers (515F and 806R) contained adapter sequences for Illumina sequencing as well as Golay barcodes (Caporaso *et al.* 2012) on the forward primer. Each PCR reaction contained 9.5 µL of MO BIO PCR Water (Certified DNA-Free), 12.5 µL of QuantaBio’s AccuStart II PCR ToughMix, 200 pM of each primer, and 1 µL of DNA (or more if the initial amplification failed). The PCR cycling conditions were: 1) initial denature at 94°C for 3 minutes, 2) 35 cycles of 94°C for 45 s, 50°C for 60 s, and 72°C for 90 s, and 3) a final hold at 72°C for 10 minutes. The three replicate PCRs were combined, and a pool was made from equimolar amounts of each sample for sequencing.

**Supplemental References**

Caporaso JG, Lauber CL, Walters WA *et al.* (2012) Ultra-high-throughput microbial community analysis on the Illumina HiSeq and MiSeq platforms. *The ISME Journal*, **6**, 1621–1624.

MO BIO Laboratories Inc (2018) PowerFecal® DNA Isolation Kit. 1–16.

Turjeman S, Corl A, Wolfenden A *et al.* (2020) Migration, pathogens and the avian microbiome: A comparative study in sympatric migrants and residents. *Molecular Ecology*, **29**, 4706–4720.

**Supplemental Tables**

| **Nest groups** | **Sample size** | **Nestbox F-value** | **Nestbox P-value** | **Dispersion P-value** |
| --- | --- | --- | --- | --- |
| 152 & 238 | 9 | 1.75 | 0.008 | 0.644 |
| 157 & 201 | 9 | 2.11 | 0.009 | 0.905 |
| 172 & 213 | 8 | 1.86 | 0.028 | 0.206 |
| 136 & 149 | 8 | 1.30 | 0.059 | 0.518 |
| 155 & 226 | 7 | 1.20 | 0.029 | 0.787 |
| 100 & 165 | 7 | 1.55 | 0.029 | 0.001 |
| 53 & 260 | 4 | 1.06 | 0.667 | 0.042 |

Supplemental Table 1: Tests of whether microbial community composition of unmanipulated pairs of nest boxes were differentiated from one another. PERMANOVA tests of Jaccard dissimilarities among nestlings were performed to evaluate differences among the nest boxes (i.e., nest box *F-*value and *P*-value). The dispersion *P-*value comes from at test of homogeneity of group dispersions.

**Supplemental Figures**


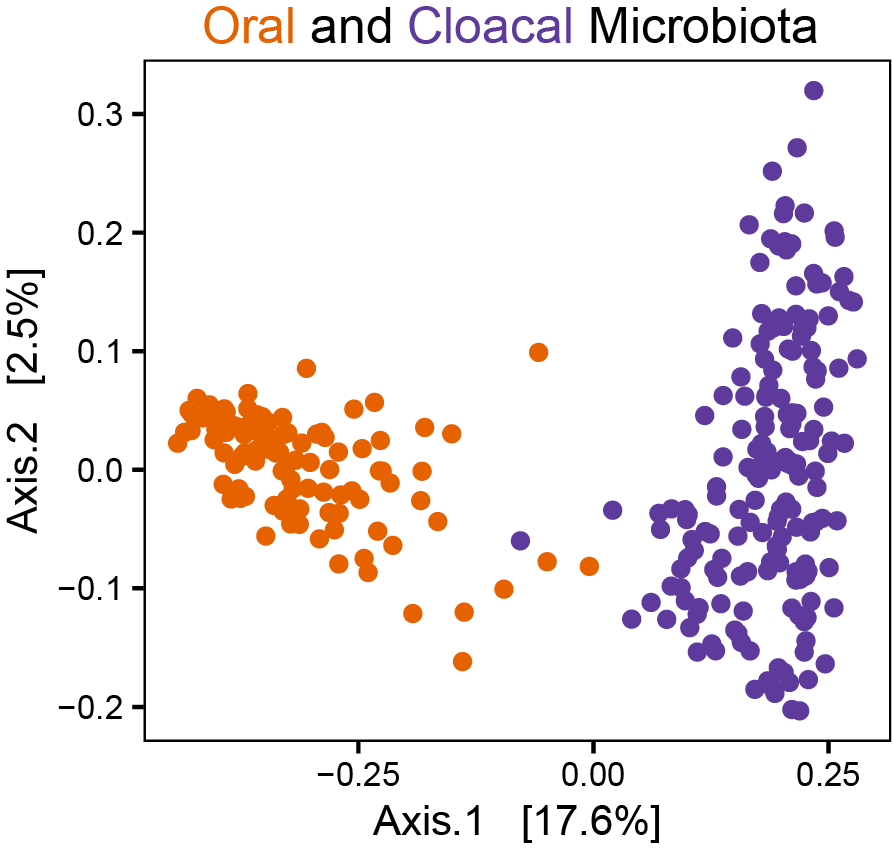


Supplemental Figure 1: Differences in the bacterial community composition for nestling oral and cloacal swabs as measured by Jaccard dissimilarity and plotted with multidimensional scaling. Oral swabs = orange points, cloacal swabs = purple points. The percentage of variation explained by each axis is given in brackets.


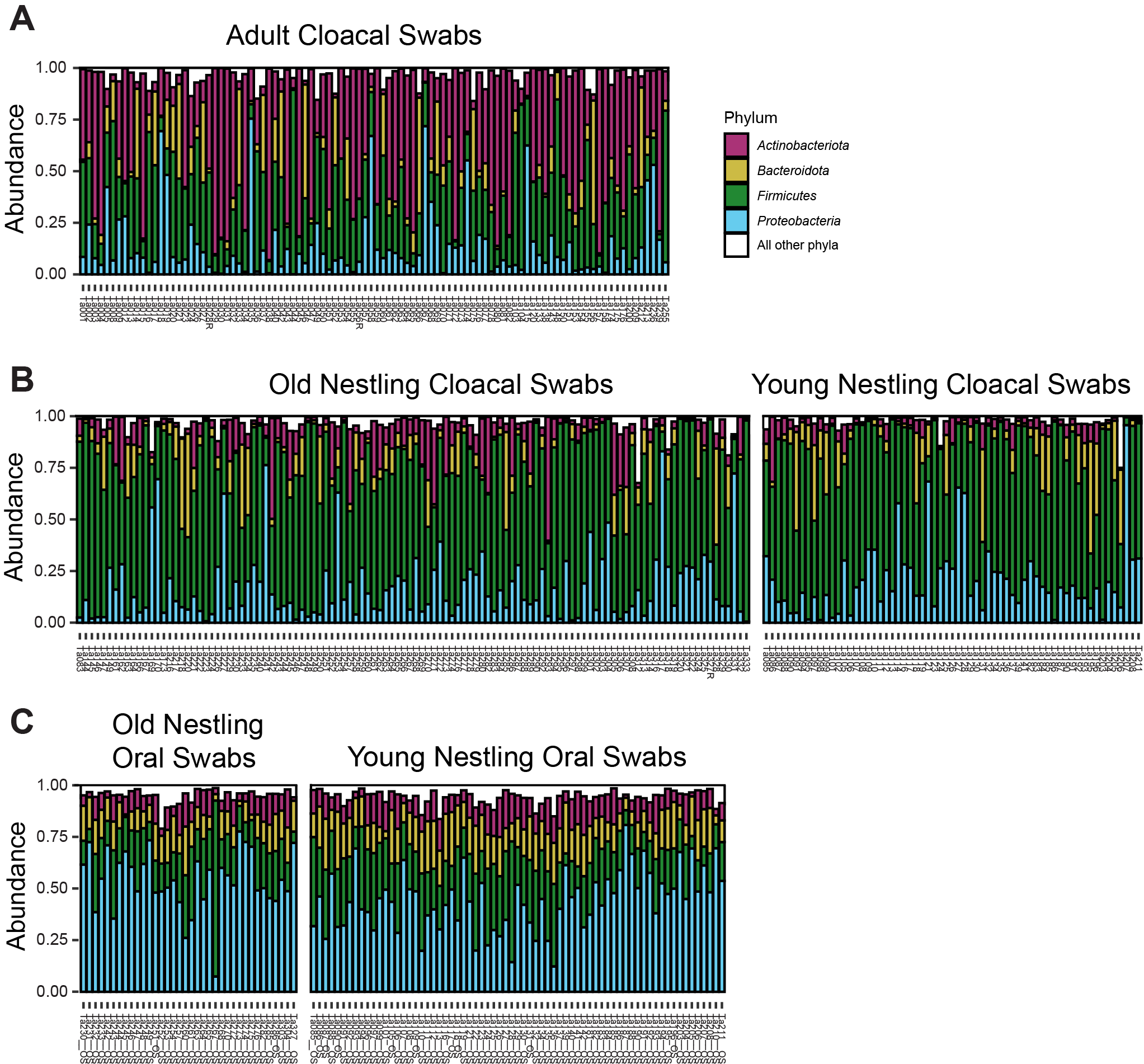


Supplemental Figure 2: Barplots for each individual owl sample depicting the top four most abundant bacterial phyla across all samples, including adult cloacal swabs (A), nestling cloacal swabs (B), and nestling oral swabs (C). Each barplot depicts the proportional abundance of the four phyla in a sample relative to the total abundance of all bacterial phyla in that sample (including additional phyla at low abundance).


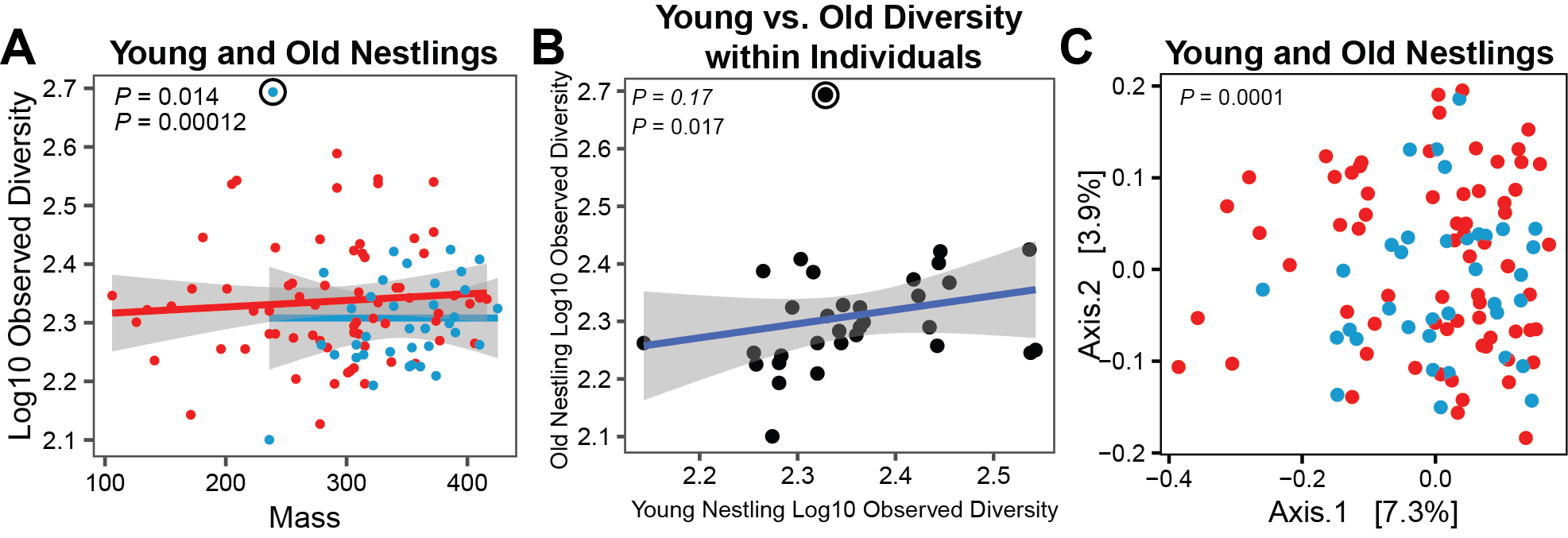


Supplemental Figure 3: Effects of age on the oral microbiota. A: Alpha diversity in relation to both the mass (g) of the individual and its age class. Trend lines with 95% confidence intervals in gray are included to enable comparisons to Figure 2, but there was not a significant correlation with mass (*P* = 0.605). B: Relationship between the oral observed diversity for young versus old nestlings within individual birds. In panels A and B, the removal of the circled outlying point results in the *P*-value changing from the top value to the bottom value. C: Changes in bacterial community composition with age as measured by Jaccard dissimilarity and plotted with multidimensional scaling with the percentage of variation explained by each axis given in brackets. Young nestlings = red points, old nestlings = blue points. *P*-values within the A and C plots are for the effect of age and in plot B it is for the correlation between the old and young observed diversity.


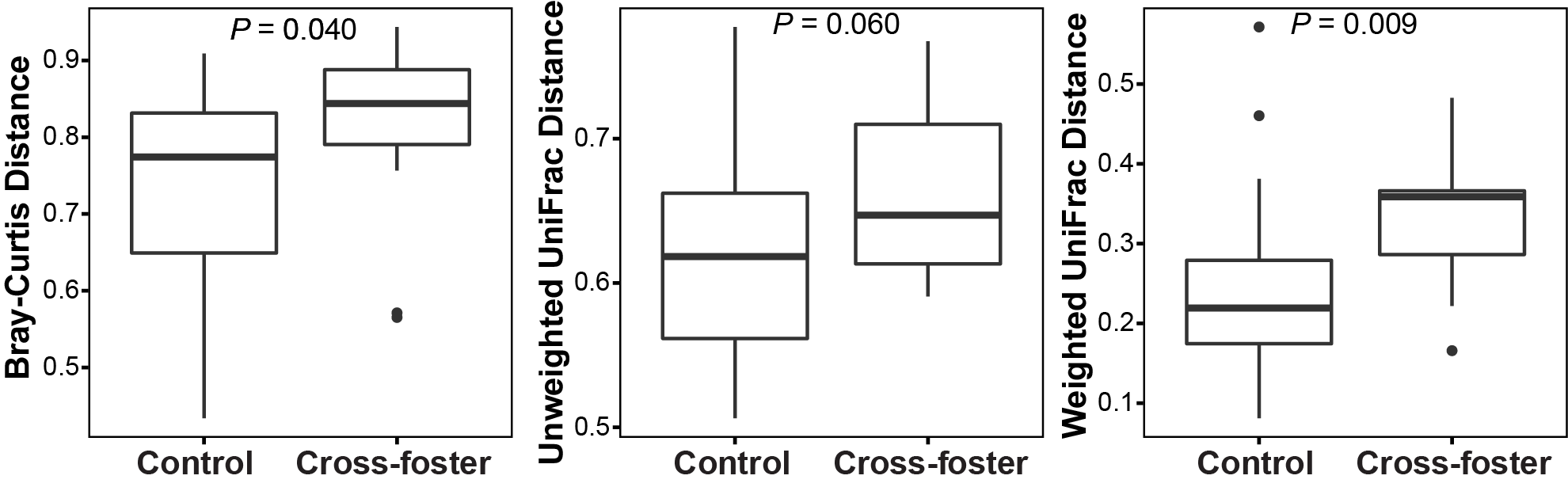


Supplemental Figure 4: Change in the microbial community composition in relation to the cross-fostering experiment. The amount of change in the microbiota for each individual nestling when it was young vs. old was measured by Bray-Curtis (left), unweighted UniFrac (middle), and weighted UniFrac (right) and then summarized across individuals with boxplots. The boxplots show the median as a thick line within boxes of the 25th and 75th percentiles, with whiskers for the minimum and maximum, and outliers as separate points.


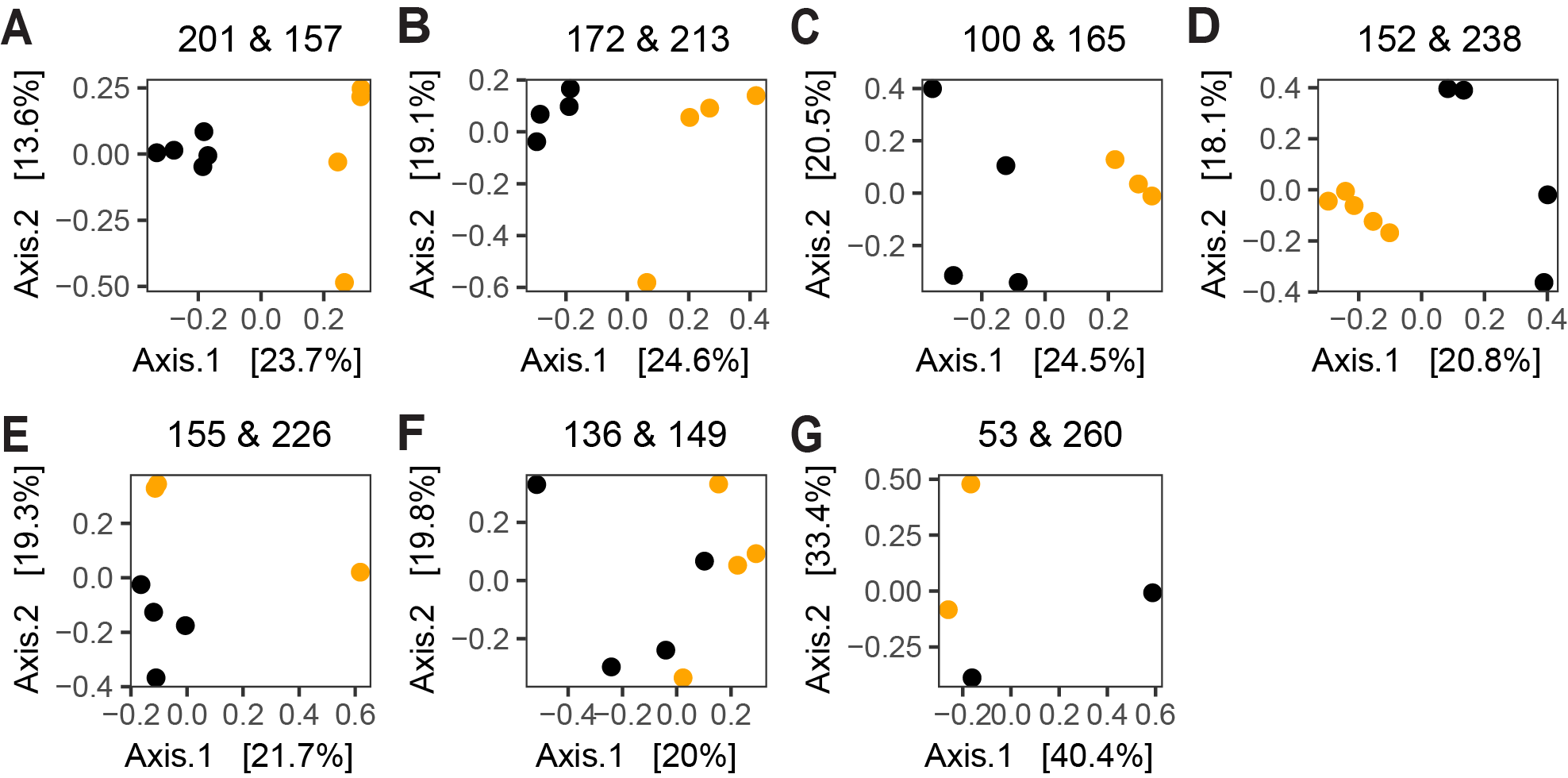


Supplemental Figure 5: The microbial community composition of pairs of unmanipulated nests with old nestlings. Nestlings are colored according to their nest of origin (orange vs. black). The Jaccard dissimilarity for each pair of nests was separately calculated and plotted with multidimensional scaling, with the percentage of variation explained by each axis given in brackets.


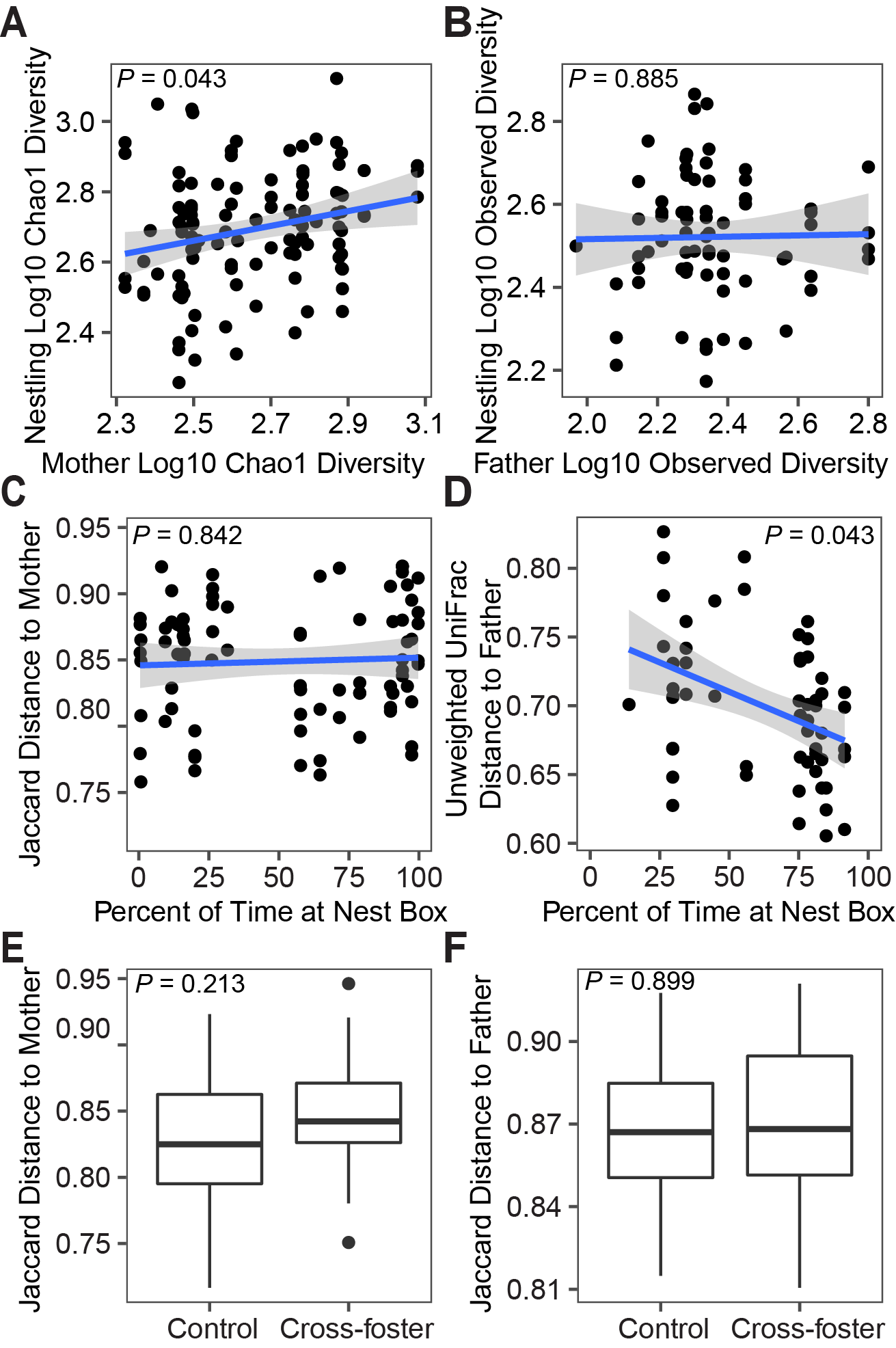


Supplemental Figure 6: Relationships between the cloacal microbiota of the nestlings with their parents. A. The relationship between the Chao1 diversity of the nestlings with Chao1 diversity of their mother. B. The relationship between the observed diversity of the nestlings with observed diversity of their father. C. Relationship between the percent of time an adult female spent at the nest and the Jaccard distance between that female's microbiota and each of her nestlings. D. Relationship between the percent of time an adult male spent at the nest and the unweighted UniFrac distance between that male's microbiota and each of his nestlings. E and F. The Jaccard distance between nestlings and their mother (E) and father (F) summarized across individuals with boxplots for the two conditions in the cross-fostering experiment. The *P*-values in panels E and F are from tests of whether the experimental conditions differ from one another. Note that while we have generally standardized our plots in the paper to show observed diversity and Jaccard distances, panels A and D show different metrics. We use Chao1 diversity in panel A because it has a statistically significant relationship compared to when observed diversity was used (Figure 6A, *P* = 0.066). Similarly, we use unweighted UniFrac distance in panel D because it has a statistically significant relationship compared to Jaccard distance (Figure 6C, *P* = 0.062). Panels A-D include linear trend lines along with 95% confidence intervals in gray. The boxplots in panels E and F show the median as a thick line within boxes of the 25th and 75th percentiles, with whiskers for the minimum and maximum, and outliers as separate points.
